# Circulating tumor cell plasticity determines breast cancer therapy resistance via neuregulin 1–HER3 signaling

**DOI:** 10.1038/s43018-024-00882-2

**Published:** 2025-01-03

**Authors:** Roberto Würth, Elisa Donato, Laura L. Michel, Massimo Saini, Lisa Becker, Tasneem Cheytan, Daria Doncevic, Tobias Messmer, Ewgenija Gutjahr, Rebecca Weber, Corinna Klein, Hamed Alborzinia, Umut Yildiz, Vanessa Vogel, Mario Hlevnjak, Polina Kozyulina, Sarah-Jane Neuberth, Paul Schwerd-Kleine, Sevinç Jakab, Nicole Pfarr, Arlou Kristina Angeles, Astrid K. Laut, Darja Karpova, Mattia Falcone, Olaf Hardt, Benjamin Theek, Celina V. Wagner, Mirjam Becker, Sabine Wagner, Martina Haselmayr, Anita Schmitt, Carsten Müller-Tidow, Sabine Riethdorf, Klaus Pantel, Marc Zapatka, Holger Sültmann, Carl Herrmann, Verena Thewes, Peter Lichter, Andreas Schneeweiss, Martin R. Sprick, Andreas Trumpp

**Affiliations:** 1 Division of Stem Cells and Cancer, German Cancer Research Center (DKFZ) and DKFZ–ZMBH Alliance, Heidelberg, Germany; 2 Heidelberg Institute for Stem Cell Technology and Experimental Medicine (HI-STEM gGmbH)), Heidelberg, Germany; 3 Gynecologic Oncology, National Center for Tumor Diseases (NCT), University of Heidelberg and German Cancer Research Center (DKFZ), Heidelberg, Germany; 4 Department of Bioinformatics, Institute of Pharmacy and Molecular Biotechnology & BioQuant, Heidelberg University, Heidelberg, Germany; 5 Institute of Pathology, University Hospital Heidelberg, Heidelberg, Germany; 6 Computational Oncology, Molecular Precision Oncology Program, German Cancer Research Center (DKFZ) and National Center for Tumor Diseases (NCT), Heidelberg, Germany; 7 Institute of Pathology, TUM School of Medicine and Health, Technical University of Munich, Munich, Germany; 8 Division of Cancer Genome Research, German Cancer Research Center, National Center for Tumor Diseases, Heidelberg, Germany; 9 Miltenyi Biotec, Bergisch Gladbach, Germany; 10 Department of Internal Medicine V, University Hospital Heidelberg, Heidelberg, Germany; 11 Department of Tumor Biology, University Medical Center Hamburg-Eppendorf, Hamburg, Germany; 12 Division of Molecular Genetics, German Cancer Research Center (DKFZ), Heidelberg, Germany; 13 German Cancer Consortium (DKTK), Heidelberg, Germany; 14 National Center for Tumor Diseases (NCT) Heidelberg, a partnership between DKFZ and Heidelberg University, Heidelberg, Germany

**Keywords:** Breast cancer, Mechanisms of disease, Cancer models, Cancer

## Abstract

Circulating tumor cells (CTCs) drive metastasis, the leading cause of death in individuals with breast cancer. Due to their low abundance in the circulation, robust CTC expansion protocols are urgently needed to effectively study disease progression and therapy responses. Here we present the establishment of long-term CTC-derived organoids from female individuals with metastatic breast cancer. Multiomics analysis of CTC-derived organoids along with preclinical modeling with xenografts identified neuregulin 1 (NRG1)–ERBB2 receptor tyrosine kinase 3 (*ERBB3*/HER3) signaling as a key pathway required for CTC survival, growth and dissemination. Genome-wide CRISPR activation screens revealed that fibroblast growth factor receptor 1 (FGFR1) signaling serves a compensatory function to the NRG1–HER3 axis and rescues NRG1 deficiency in CTCs. Conversely, NRG1–HER3 activation induced resistance to FGFR1 inhibition, whereas combinatorial blockade impaired CTC growth. The dynamic interplay between NRG1–HER3 and FGFR1 signaling reveals the molecular basis of cancer cell plasticity and clinically relevant strategies to target it. Our CTC organoid platform enables the identification and validation of patient-specific vulnerabilities and represents an innovative tool for precision medicine.

## Abstract (web summary)

Trumpp and colleagues develop a method to obtain long-term circulating tumor cell-derived organoids from individuals with metastatic breast cancer and identify the neuregulin 1–HER3 axis as important for organoid growth and a promising therapeutic target.

## Main

Breast cancer (BC) treatment and prognosis have been greatly improved in recent years. Nevertheless, metastatic BC (MBC) remains incurable. BC cells spread mainly to the lungs, liver, bones, lymph nodes and brain, and the source of these metastases has been suggested to be circulating tumor cells (CTCs) with metastasis-initiating ability^1^. Even though CTC enumeration serves as a prognostic marker^2,3^, CTC biology remains poorly understood^4^. Notably, the field still lacks a standardized method for the functional characterization of CTCs, which is essential to unveil the mechanisms and pathways involved in tumor spreading, dissemination and colonization to distant organs. The scarcity of CTCs in blood and the difficulty of culturing them limit their use for functional analyses. Limited publications have reported the possibility of expanding CTCs from few patients with MBC (MBCPs) with extremely high CTC numbers^5–7^. Nonetheless, a systematic and detailed assessment of the in vitro conditions required to generate long-term propagatable CTCs is lacking^8^. Liquid biopsies are easy to obtain and almost noninvasive, and they provide a continuous source of tumor-derived material (CTCs or circulating cell-free tumor DNA), granting the possibility of longitudinal sampling. The establishment of experimental platforms supporting reliable isolation and expansion of CTCs is therefore of great interest due to relevant clinical implications^9^, including the identification of adaptive resistance mechanisms and CTC plasticity in response to environmental challenges^10,11^.

A recent study suggested that CTC lifespan is the most critical parameter governing metastasis^12^. Hence, identification of the pathways responsible for CTC survival represents a crucial step for blocking CTC dissemination. The treatment algorithms for MBC are mainly decided based on estrogen receptor (ER), progesterone receptor and ERBB2 receptor tyrosine kinase 2 (*ERBB2*/HER2) expression, assessed by immunohistochemistry (IHC) staining of the primary tumor. To date, HER2 inhibitors, such as trastuzumab, pertuzumab, tucatinib, lapatinib and HER2-directed antibody–drug conjugates (such as trastuzumab deruxtecan), are the standard approaches exclusively for BC with *HER2* amplification even though HER2-expressing cells also exist in HER2^–^/HER2^lo^ BCs^13,14^. Indeed, a recent landmark study demonstrated that treatment with trastuzumab deruxtecan significantly prolonged progression-free survival in individuals with HER2^lo^ BC^15^, highlighting the relevance of the HER2 pathway in MBC irrespective of the HER2 IHC score determined at diagnosis. HER2 activates downstream oncogenic signaling via the PI3K–AKT, MAPK and JAK–STAT pathways, mainly by dimerizing with its partner HER3 (ref. ^16^), which contains the binding pocket for the ligand neuregulin 1 (NRG1). Although the NRG1–HER3 pathway has been studied in BC^17^ and other entities^18^, only limited information is available on its role in MBC CTCs.

Here, we established a method for the long-term expansion of MBCP-derived CTCs from multiple liquid biopsy sources. Using this platform, we identified NRG1 as a key factor that promotes HER3^+^ CTC survival, facilitating metastatic growth. Moreover, we uncovered FGFR1 signaling as a compensatory mechanism able to sustain CTC survival and growth in the absence of NRG1. Last, we provide functional data showing that combinatorial blockade of NRG1 and FGFR1 signaling could efficiently target CTCs in MBCPs.

## Results

### Role of NRG1 in metastasis and CTC-derived organoid establishment

**Fig. 1:**
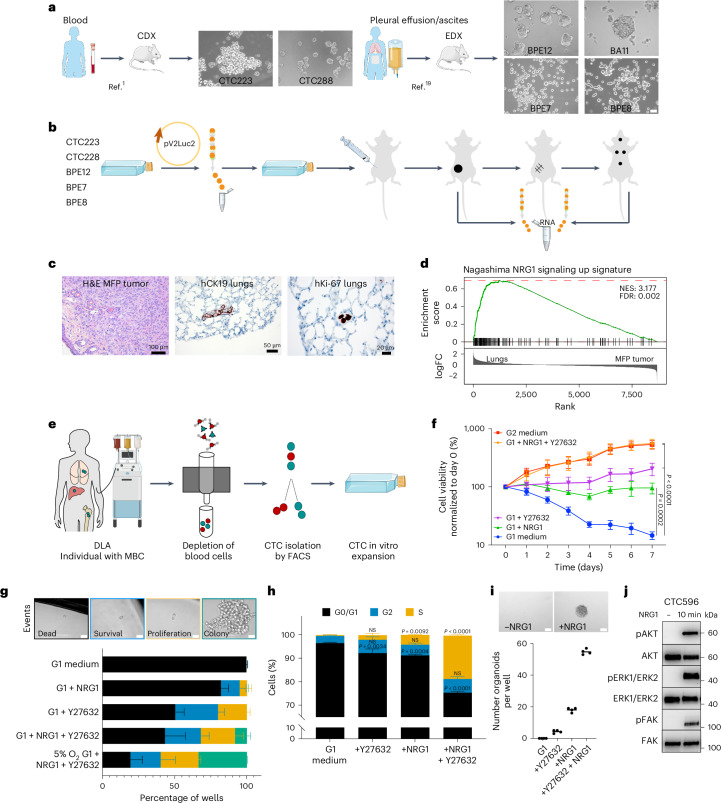
NRG1 signaling is upregulated in metastasis-initiating cells in vivo and is crucial to establish primary CDOs. **a**, CDX and EDX in vitro models. Scale bar, 20 µm. BPE, pleural effusion from a patient with breast cancer; BA peritoneal effusion from a patient with breast cancer **b**, In vivo preclinical model of spontaneous metastatic formation. **c**, Representative IHC images of mammary fat pad (MFP) tumor (hematoxylin and eosin (H&E)) and matched lung-colonizing cells detected using human-specific antibodies to CK19 and Ki-67; experiments were repeated three times independently with similar results. **d**, Gene set enrichment analysis (GSEA) applying the ‘Nagashima NRG1 signaling up’ signature^21^ to the dataset of sorted lung-colonizing cells (Lungs) and matched primary tumor cells (MFP tumor); NES, normalized enrichment score; FDR, false discovery rate; FC, fold change. **e**, CTC isolation and in vitro direct expansion workflow. **f**, CTC596 cell growth under different medium conditions using the CellTiter Blue (CTB) assay. Mean values were normalized to day 0 for each condition and log_10_ transformed to enhance approximate normality. Data are shown as mean ± s.e.m.; *n* = 3 biological replicates for G1 + Y27632 and G1 + NRG1 + Y27632; *n* = 4 biological replicates for G1, G1 + NRG1 and G2. Data were analyzed by two-way analysis of variance with a Dunnett’s multiple comparisons test; G1 + NRG1, *P* = 0.0002; G1 + Y27632, G1 + NRG1 + Y27632 and G2, *P* < 0.0001, each versus G1 medium at day 7. **g**, Top: clonogenic assay representative brightfield images. Scale bar, 10 μm. Bottom: stacked bar plot showing the percentages of colonies (green), proliferating cells (orange), surviving cells (blue) and dead cells (black). Data are shown as mean ± s.d.; *n* = 2 biological replicates for 5% O_2_ G1 + NRG1 + Y27632, G1 + NRG1 + Y27632; *n* = 3 biological replicates for G1 + Y27632, G1 + NRG1 and G1. **h**, Cell cycle phase distribution (S (orange), G2 (blue) and G0/G1 (black)) after 48 h under different medium conditions. Data are shown as mean ± s.e.m.; *n* = 3 biological replicates. Data were analyzed by two-way ANOVA test with a Dunnett’s multiple comparisons test versus G1; NS, not significant; G0/G1: +Y27632, *P* = 0.0034; +NRG1, *P* = 0.0004; +NRG1 + Y27632, *P* < 0.0001. S: +NRG1, *P* = 0.0092; +NRG1 + Y27632, *P* < 0.0001. **i**, Top: representative brightfield images of CTC596 organoids without (left) or with (right) NRG1 (20 ng ml^–1^). Scale bars, 100 μm. Bottom: organoid quantification. Bars represent the mean, and each dot represents a technical replicate (*n* = 4). **j**, Representative western blot showing phosphorylated and total levels of AKT, ERK1/ERK2 and FAK, with or without NRG1 (20 ng ml^–1^, 10 min). Experiments were repeated two times independently with similar results. Source data

**Extended Data Fig. 1:**
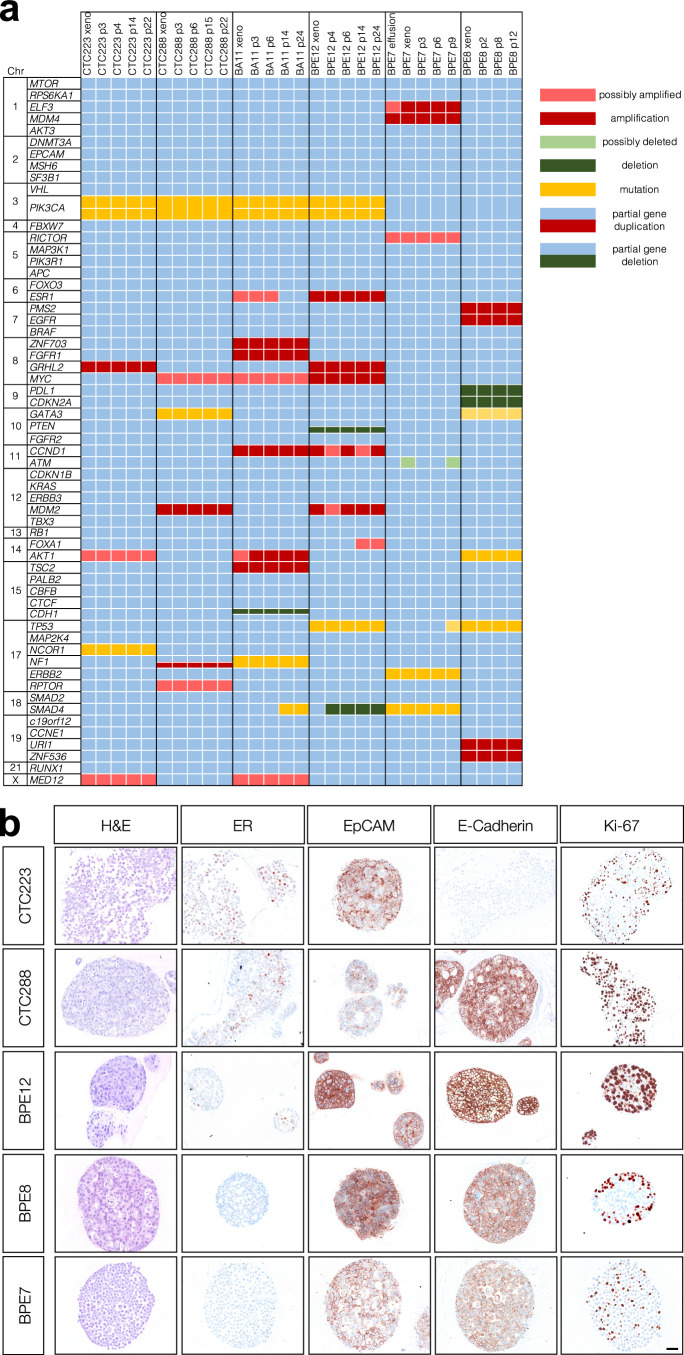
Characterization of CDX and EDX-derived organoids. **a**. Oncoprint showing the molecular alterations including the most frequent mutated genes in breast cancer. Targeted-DNA panel sequencing has been performed on longitudinal CDX and EDX-derived CDOs and from the xenograft they have been generated (labelled as ‘xeno’). Different *in vitro* passages of xenograft-derived CDOs are labelled with p and the number. Pink: possibly amplified; Red: amplified; Light green: possibly deleted, Green: deleted; Mustard: mutation; Azure/Red: partial gene duplication; Azure/Green: partial gene deletion. **b**. Representative IHC pictures of different CDX- and EDX-derived CDOs, repeated two times independently with similar results. Scale bar 50 µm.

In the initial attempt to establish in vitro conditions for MBCP-derived CTCs, we pre-expanded cells in vivo, generating xenografts where CTCs (CTC-derived xenograft (CDX))^1^ or cells collected from effusions (effusion-derived xenograft (EDX))^19^ were transplanted into immunocompromised NOD.Cg-*Prkdcscid Il2rgtm1Wjl/*SzJ (NSG) mice. We then isolated tumor cells from the xenografts and expanded them in a serum-free molecularly defined medium containing different supplements that support the organotypic growth of primary cells (first-generation G1 medium). CTC-derived organoids (CDOs) were expanded as three-dimensional structures, either in a matrix-free condition or embedded in a collagen-based chemically defined floating matrix^20^. Using this strategy, we successfully established and expanded a total of six different CDX or EDX models for more than 20 passages while preserving their genetic profile (Fig. 1a and Supplementary Table 1). Only in one out of six models did cells acquire an additional mutation in *SMAD4* at passage 14, with concomitant change in their morphology (Extended Data Fig. 1a). Importantly, the CDOs faithfully recapitulated the original individual and matched xenograft subtypes (Extended Data Fig. 1b). Thus, we were able to establish human BC CDOs from CDXs and EDXs while maintaining patient-specific characteristics.

**Extended Data Fig. 2:**
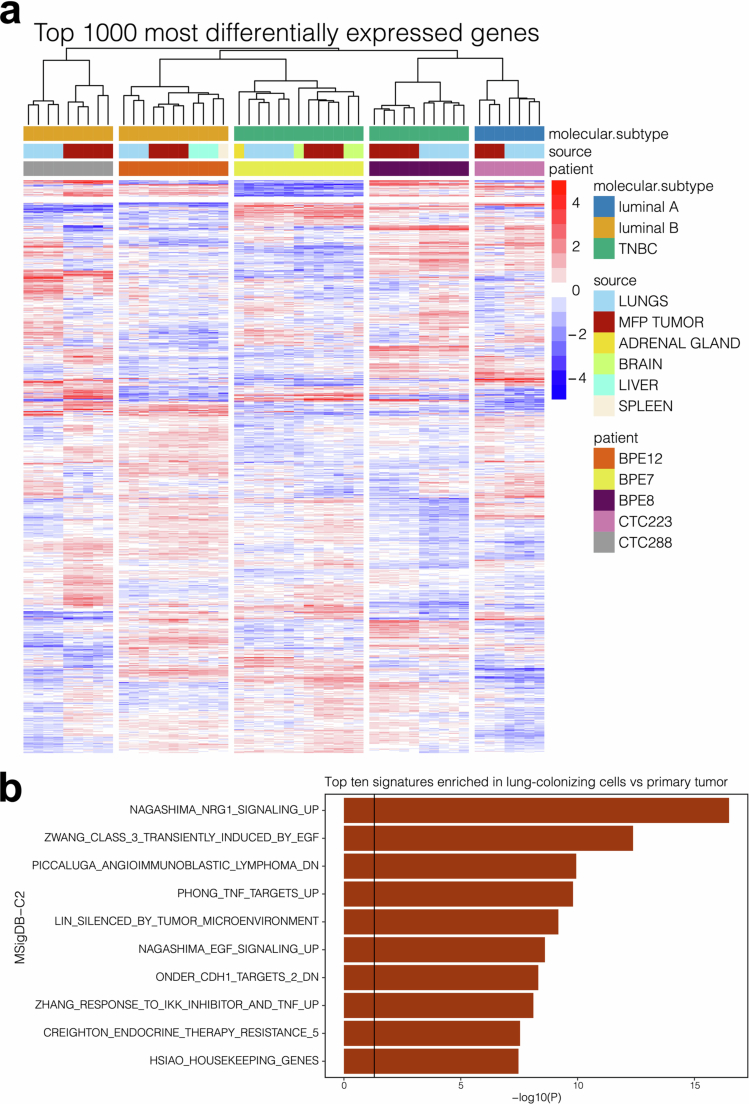
Neuregulin 1 signaling is up-regulated in metastasis-initiating cells *in vivo.* **a**. Heatmap showing the expression levels of the top 1000 most differentially expressed genes. Within each patient, transcriptional profiles clustered based on the sample source: primary tumor ´MFP tumor´ or different metastatic sites: lungs, adrenal gland, brain, liver, spleen. **b**. Top ten signatures enriched in metastatic cells, using as dataset the sorted lung-colonizing cells (Lungs) and matched cells from the primary tumor (MFP Tumor), ranked according to the -log10 p value (one side) after gene set enrichment analysis (GSEA) with *gage* R package using the C2 curated gene sets from MSigDB.

**Extended Data Fig. 3:**
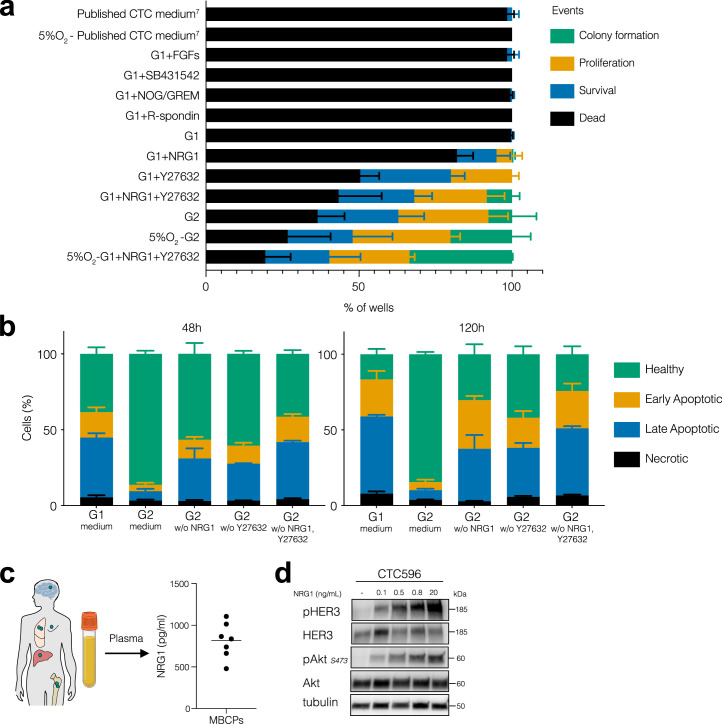
CTC medium and NRG1 in plasma. **a**. Stacked bar plots showing the percentages of growing colonies (green), proliferating cells (orange), surviving cells (blue) and dead cells (black) in a clonogenic assay. Different media conditions are used (see Material and Methods for more details on media composition). Counting was performed 30 days after single CTC596 cells FACS sorting. Data are shown as mean ± s.d., n = 2 5%O_2_ G1 + NRG1 + Y27632, G1 + NRG1 + Y27632, G1 + R-spondin, G1+Noggin (NOG), Gremlin-1 (GREM), G1 + SB431542, G1+FGFs, 5%O_2_ Published medium, Published medium, n = 3 5%O_2_ G2, G2, G1 + Y27632, G1 + NRG1, G1 biological replicates. **b**. Stacked bar plots showing live or dead CTC596 cell distribution (healthy (green), early apoptotic (orange), late apoptotic (blue), and necrotic (black)) after 48 (left) and 120 (right) hours in either G1, G2, G2 w/o NRG1, G2 w/o Y27632, or G2 w/o both Y27632 and NRG1 medium. Cell state analysis was determined via flow cytometry after Annexin and Phosphoinositol (PI) staining. Data are mean ± s.e.m, n = 3 biological replicates. two-way ANOVA test, Dunnett´s multiple comparisons test, statistical analysis is reported in Source Data. **c**. Plot showing NRG1 concentration (pg/ml) in blood samples of metastatic breast cancer patients (MBCPs, n = 7) measured with the Human NRG1 ELISA kit. **d**. Western blot analysis of whole-cell protein lysate derived from CTC596 cells in absence or presence of NRG1 at different concentrations (0.1, 0.5, 0.8 and 20 ng/ml), including the average NRG1 concentration in the blood of MBC patients (0.8 ng/ml). Phosphorylated and total HER3 and Protein Kinase B (Akt) were detected, tubulin was used as the loading control, n = 1. Source data

To avoid the time-consuming patient-derived xenograft (PDX) pre-expansion step and thus be able to analyze tumor cells in the laboratory in parallel to the clinical course of disease, we tested the efficacy of the G1 medium in supporting the expansion of CTCs directly isolated from MBCP liquid biopsies. G1 was not sufficient to promote cell proliferation nor to maintain cell survival of CTCs in vitro. Therefore, to optimize culture conditions for primary CDOs, we performed transcriptomic analysis on primary and metastatic cells from preclinical xenografts with the aim of identifying the molecular pathways critical for CTC growth in vivo (Fig. 1b). To study the early stages of cell spreading and distant organ colonization after primary tumor removal, we analyzed the micrometastatic stage whereby only small clusters of cells were present (Fig. 1c). As expected, interparticipant heterogeneity was larger than the intraparticipant differences between primary and metastatic cells (Extended Data Fig. 2a). Within each individual, primary tumors clustered separately from metastatic cells, suggesting substantial transcriptional differences already at the micrometastatic stage. As all the models consistently gave rise to lung metastases while other organs were differentially colonized, we focused on lung-metastatic cells. Here, we found an NRG1-dependent signature^21^ that was strongly enriched in lung-colonizing cells compared to in the primary tumor (Fig. 1d and Extended Data Fig. 2b). Based on these results, we supplemented the G1 medium with NRG1 (20 ng ml^–1^) along with other factors that are beneficial for ex vivo carcinoma cultures, namely FGF3, FGF7, FGF8, FGF9, FGF10, noggin, gremlin-1, SB431542 (activin–BMP–TGFβ pathway inhibitor) and Y27632 (ROCK inhibitor)^22,23^. To test this now-termed G2 formula, we established a protocol to isolate CTCs directly from MBCP blood by diagnostic leukapheresis (DLA; CTC596). The DLA product was processed by depletion of hematopoietic and endothelial cells, followed by enrichment of EPCAM^+^CD45^−^ CTCs by fluorescence-activated cell sorting (FACS; Fig. 1e and Supplementary Fig. 1). Both our G1 medium and previously reported CTC medium^7^ were not sufficient to promote cell proliferation nor to maintain CTC survival. By contrast, G2 medium allowed the exponential growth of CDOs over time, with a significant reduction of apoptotic and necrotic cells compared to CDOs in the original G1 medium (Fig. 1f,g and Extended Data Fig. 3a,b).

To identify the essential supplements required and sufficient for the robust proliferative effect of G2 medium, each molecule/growth factor was tested separately by either the addition to G1 medium or removal from G2 medium, respectively. Remarkably, although none of the single growth factors tested was able to completely recapitulate the effects observed using G2, NRG1 and Y27632 promoted survival and proliferation when added individually to the G1 medium (Fig. 1h,i). Strikingly, by adding both NRG1 and Y27632, a synergistic effect on CDO growth was observed, and the addition of both compounds fully phenocopied the growth mediated by G2 (Fig. 1f–h and Methods). We therefore defined ‘CTC medium’ as G1 + NRG1 + Y27632. Interestingly, we observed that low oxygen (5%) enhanced cell viability and colony formation (Fig. 1g), similar to what was observed for induced pluripotent stem cells^24^. Finally, we confirmed that NRG1 treatment triggered downstream signaling pathways, including AKT, MAPK and FAK (Fig. 1j).

These data suggest that CTC survival in blood may be supported by the presence of NRG1 in the bloodstream. We therefore measured NRG1 levels in plasma from MBCP blood samples (*n* = 7) and detected an average concentration of 815.14 pg ml^–1^ (range of 479–1,107 pg ml^–1^; Extended Data Fig. 3c). These NRG1 levels are likely sufficient to activate the downstream signaling pathways because as low as 100 pg ml^–1^ was sufficient to phosphorylate and activate HER3 and AKT in CTC596 cells (Extended Data Fig. 3d).

Together, our data demonstrate that NRG1 signaling plays a key role in the establishment and growth of primary CTCs in vitro.

### Long-term CDOs from individuals with MBC

**Fig. 2:**
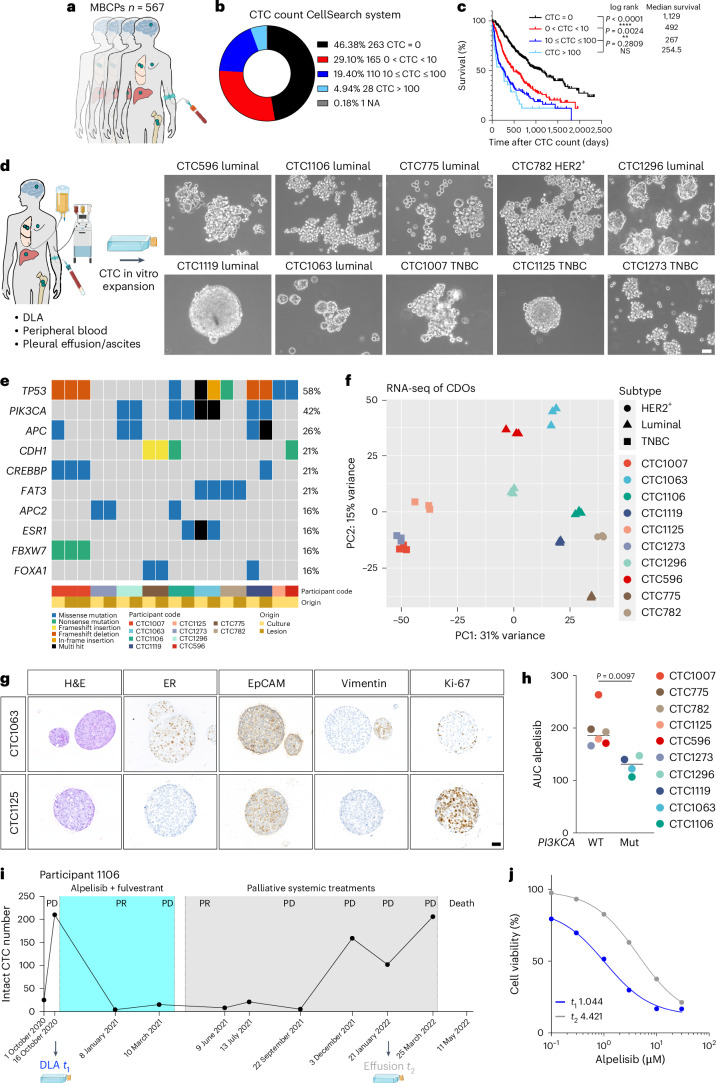
Systematic establishment and characterization of long-term CDOs directly from individuals with MBC. **a**, MBCP cohort (*n* = 567). **b**, Distribution of participants according to CTC number per 7.5 ml of blood using the CellSearch system (Menarini). NA, not available. **c**, Probability of survival stratified by CTC count in MBCPs (*n* = 566 participants). Data were analyzed by log rank (Mantel–Cox) test, and the *P* value of each comparison is reported. **d**, Left: isolation of CTCs from peripheral blood or effusion products. Right: brightfield images of the CDOs are shown. Scale bar, 50 μm. **e**, Mutational profile of the top ten most commonly mutated genes in CDOs and matched participant lesions. **f**, PCA plot using RNA-seq data from CDOs. Different tumor subtypes are indicated with different shapes (HER2^+^, circle; luminal, triangle; TNBC, square). RNA-seq libraries were prepared in triplicates for each model. **g**, Representative IHC images for H&E, ER, EpCAM, vimentin and Ki-67 protein expression in CDOs from the CTC1063 luminal subtype and CTC1125 TNBC subtype; *n* = 1; scale bar, 50 µm. **h**, Area under the curve (AUC) values for dose–response to alpelisib. A CTB assay was performed after 72 h. Bars represent the median, and each dot represents the AUC mean derived from two independent experiments for each CDO. CDOs were grouped according to *PIK3CA* status: WT (left, *n* = 6) and mutated (mut; right, *n* = 4) CDOs. Data were analyzed by two-tailed unpaired *t*-test; *P* = 0.0097. **i**, CTC numbers in 7.5 ml of blood in longitudinal samples from participant CTC1106. An overview of the treatment regimen is reported; PD, progressive disease; PR, partial response. Dates are shown along the *x* axis. **j**, Plot representing the dose–response of CTC1106 (blue, *t*_1_) and CTC1106 effusion (gray, *t*_2_) CDOs to alpelisib treatment. A CTB assay was performed after 72 h. The CTB fluorescence value was normalized to the viability of cells without the drug. Dots represent the mean; *n* = 2 biological replicates. The average half-maximal inhibitory concentration (IC_50_) values are reported. Source data

**Extended Data Fig. 4:**
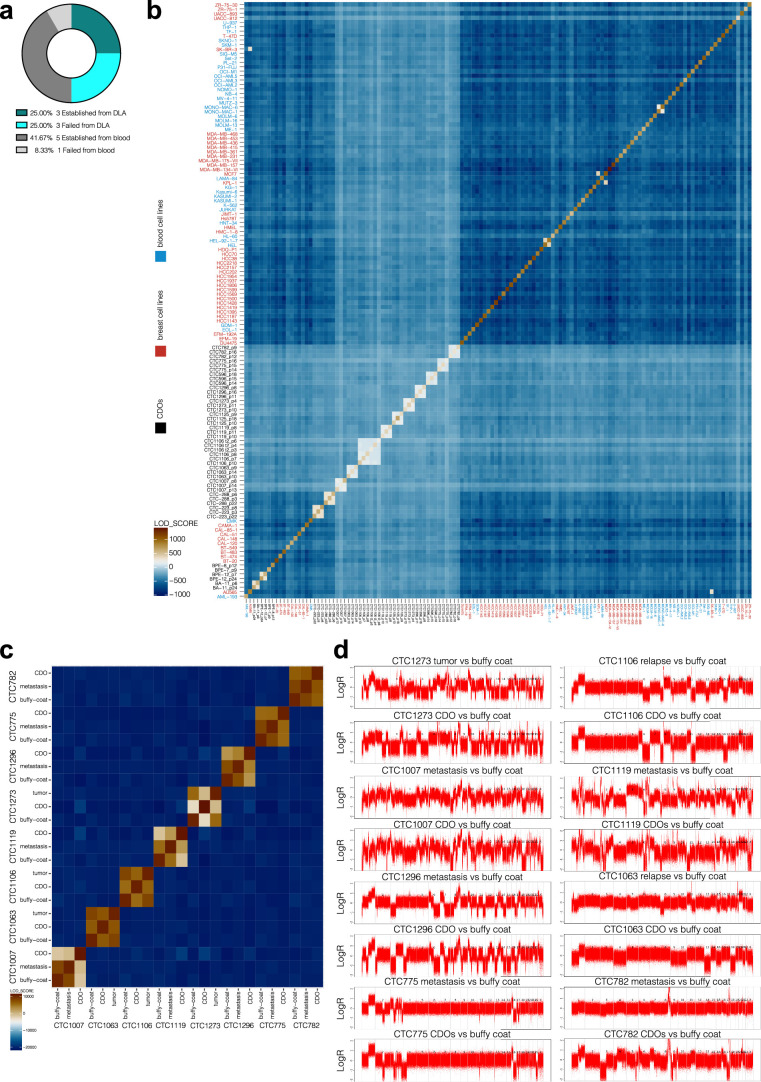
Establishment and characterization of long-term CDOs. **a**. Donut plot showing successful (Establishment) or unsuccessful (Failed) attempts to obtain long-term CDOs. **b**. Heatmap showing the logarithm of the odds (LOD) score calculated for pairwise comparisons of RNAseq data from our CDOs and publicly available cell lines (from both human breast and blood cancer). **c**. Heatmap showing the LOD score calculated for pairwise comparisons of WGS data from primary patient material (buffy coat and tumor lesions) and matched CDOs. **d**. logR plots showing copy number profiles of primary patient tumor lesions and CDOs. Matched buffy coats were used as germline controls.

**Extended Data Fig. 5:**
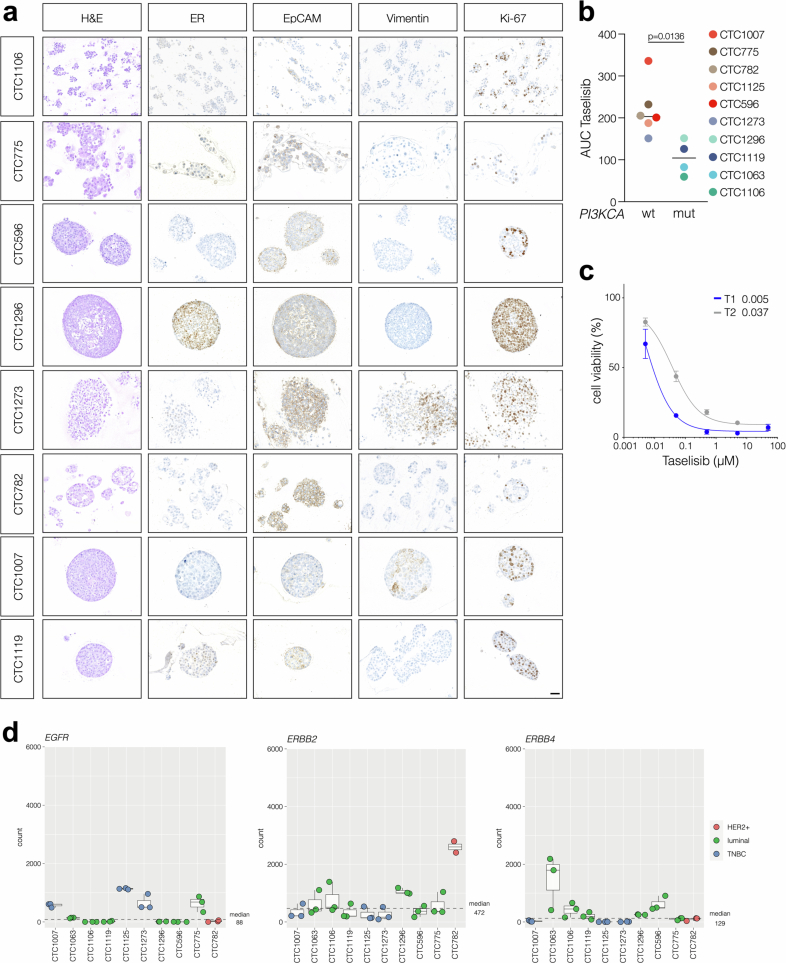
Phenotyping and drug sensitivity of long-term CDOs. **a**. Representative IHC pictures of different CDOs, n = 1. Scale bar 50 µm. **b**. Plot showing area under the curve (AUC) values for dose-response to Taselisib. Cells were grouped according to *PIK3CA* status (wild-type (wt) on the left, n = 6, mutated (mut) on the right, n = 4). CTB assay was performed after 72 hours. Each dot represents the AUC mean obtained from n = 5 CTC1106, n = 3 CTC1063, CTC1007, CTC1125, n = 2 CTC1119, CTC782, CTC775, CTC596, CTC1273, CTC1296 biological replicates. Two-tailed Unpaired t test: p = 0.0136. **c**. Plot representing the dose response of CTC1106 CDO T1 (blue) and CTC1106 CDO T2 (grey) to Taselisib. CTB assay was performed after 72 h. The CTB fluorescence value was normalized to the viability of cells without the drug. Bars represent the mean, error bars indicate standard error of the mean, n = 5 T1, n = 3 T2 biological replicates. The average IC50 values are reported (µM). **d**. Boxplots showing *EGFR*, *ERBB2*, *ERBB4* expression using RNAseq normalized counts from CDOs, n = 3 biological replicates. Boxplots show low and upper quartiles and median line is indicated. Whiskers, 1.5 × interquartile range. Dashed line represents the median expression. RNAseq libraries have been prepared in triplicates for each of the CTC models. Source data

After successful generation of the first long-term CDO, we established additional CDOs from different MBC liquid biopsy sources. To select samples with higher CTC numbers, we enumerated relatively intact CTCs in blood using the Food and Drug Administration-approved CellSearch system in a cohort of 567 MBCPs treated at the National Center for Tumor Diseases (NCT) in Heidelberg from January 2017 to April 2022 (Fig. 2a). In 263 (46.38%) samples, CTCs were not detected, 165 (29.10%) individuals showed between 1 and 9 CTCs, 110 (19.40%) individuals showed between 10 and 100 CTCs, and 28 individuals (4.94%) showed more than 100 CTCs (Fig. 2b). Survival correlated with the number of CTCs, with a median survival of 1,129 days in individuals where CTCs were not detectable, 492 days in individuals with CTC counts between 1 and 9, 267 days in individuals with CTC counts between 10 and 100 and 254.5 days in individuals with more than 100 CTCs (Fig. 2c). Next, we collected additional liquid biopsies from individuals with the highest (>100) CTC counts for CDO generation. From 8 of 12 samples (66.7%) available, we successfully generated long-term CDOs: three from DLA and five from peripheral blood (Extended Data Fig. 4a). In addition, we established CDOs from effusions from two individuals, one of which contained no CTCs in the blood. The models included all major subtypes of BC: hormone receptor^+^HER2^–^ (HR^+^HER2^–^; luminal), HR^–^HER2^–^ (triple negative BC (TNBC)) and HER2^+^ (Fig. 2d and Supplementary Table 1). The absence of cross-contamination with cell lines and the match between CDOs and the corresponding participant were confirmed (Extended Data Fig. 4b,c). CDOs were profiled for genetic alterations using participant-matched biopsies of tumor lesions as references and matched buffy coats as germline controls^25^. As expected, *TP53* and *PIK3CA* were the most frequently mutated genes, followed by *APC* and *CDH1* (Fig. 2e and Supplementary Table 2). Moreover, copy number analysis highlighted a strong consistency between the genomic profiles of CDOs and matched participant profiles (Extended Data Fig. 4d). Of note, a gain of chromosome 1q (chr1q; CTC1119 and CTC1063) or loss of chr16q (CTC1296, CTC775 and CTC1106) was observed in the matched CDOs and participant samples, in line with what was previously reported for HR^+^ BCs. In TNBC-derived CTC1273 and CTC1007 cells, a typical gain of chr10p was detected in both CDOs and matched participant samples^26^. All models were also subjected to transcriptomic analysis. Principal component analysis (PCA) showed that transcriptomes clustered primarily by participant ID, as expected. Nonetheless, the BC subtype was the main driver of PC1 separation because TNBC-derived CDOs clustered separately from either HER2^+^ or luminal-derived CDOs (Fig. 2f). CDO heterogeneity was highlighted by varying levels of EpCAM, vimentin, Ki-67 and ER expression (Fig. 2g and Extended Data Fig. 5a). Next, we demonstrated that the CDOs can be used as a clinically relevant platform to model drug sensitivity or resistance. Indeed, sensitivity to the selective PIK3CA inhibitors alpelisib or taselisib^27,28^ correlated with *PIK3CA* status (Fig. 2h and Extended Data Fig. 5b). We then collected a second liquid biopsy from participant CTC1106 (Fig. 2i) who, after an initial response, developed resistance to alpelisib/fulvestrant as assessed by computed tomography scans, tumor markers and reduced general condition. The second established CDO from CTC1106 at time point *t*_2_ showed lower sensitivity to either alpelisib or taselisib than *t*_1_ CDOs (Fig. 2j and Extended Data Fig. 5c), recapitulating the clinical resistance status of the participant at this stage.

Together, we generated 11 primary CDOs from liquid biopsies of 10 MBCPs. These CDOs serve as a platform for studying how cancer cells become resistant to therapy over time.

### HER3 is crucial for CTC growth and survival in vivo

**Fig. 3:**
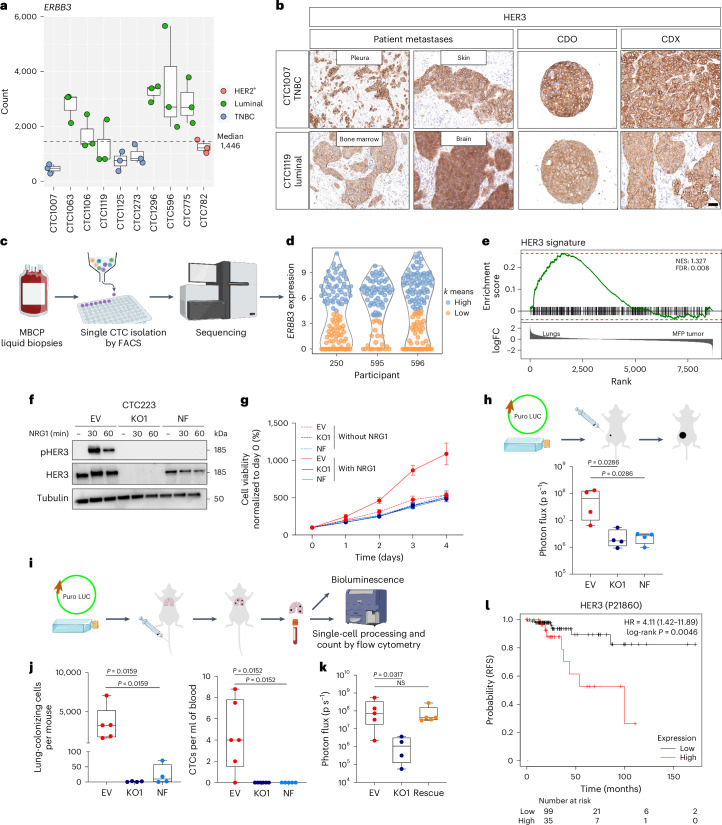
HER3 expression is crucial for CTC growth and survival in vivo*.* **a**, *ERBB3* expression; *n* = 3 biological replicates. Box plots show the median and top and bottom quartiles. Whiskers denote 1.5× the interquartile range. **b**, Representative IHC images of HER3; *n* = 1; scale bar, 50 µm. **c**, scRNA-seq workflow. **d**, *ERBB3* expression in CD45^–^EpCAM^+^ CTCs. *ERBB3*^hi^, turquoise; *ERBB3*^lo^, orange. **e**, GSEA using the HER3 CTC signature on the dataset from Fig. 1b. **f**, Western blot showing phosphorylated and total levels of HER3 with or without NRG1 (20 ng ml^–1^; 30 and 60 min) in *ERBB3*-WT or *ERBB3*-KO CTC223 cells; *n* = 1. **g**, CTB assay with (solid line) or without (dotted line) NRG1. Mean values were normalized to day 0 for each condition. Error bars indicate s.e.m.; *n* = 4 biological replicates for all conditions but EV without NRG1 day 4 and EV with NRG1 days 2, 3 and 4 (*n* = 3 biological replicates). Data were analyzed by two-way ANOVA with a Tukey’s multiple comparisons test. **h**, Top: in vivo tumorigenic assay workflow. Puro, puromycin resistance; LUC, luciferase. Bottom: tumor growth quantification via bioluminescence. Each dot represents a mouse; *n* = 4 per condition. Box plots show the median and top and bottom quartiles, and whiskers indicate minimum and maximum values. Data were analyzed by two-tailed Mann–Whitney test; KO1 versus EV, *P* = 0.0286; NF versus EV, *P* = 0.0286. **i**, In vivo metastasis assay workflow. **j**, Box plot showing tumor cell number per mouse (left, EV, *n* = 5 mice; KO1 and NF: *n* = 4 mice) and CTC number per ml of blood (right, EV and KO1, *n* = 6 mice; NF, *n* = 5 mice). Each dot represents a mouse. Box plots show median values and top and bottom quartiles, and whiskers indicate minimum and maximum values. Data were analyzed by two-tailed Mann–Whitney test (left, KO1 versus EV, *P* = 0.0159; NF versus EV, *P* = 0.0159; right, KO1 versus EV, *P* = 0.0152; NF versus EV, *P* = 0.0152). **k**, Box plot showing ex vivo lung bioluminescence intensity. Each dot represents a mouse. Box plots show median values and top and bottom quartiles, and whiskers indicate minimum and maximum values (EV and NF, *n* = 5 mice; KO1, *n* = 4 mice). Data were analyzed by two-tailed Mann–Whitney test (KO1 versus EV, *P* = 0.0317; *ERBB3* rescue versus EV, *P* = 0.8413). **l**, Relapse-free survival (RFS) in ER^+^ stage 3 BC according to HER3 protein expression interrogating TCGA RPPA cohort; low, black; high, red; HR, hazard ratio. Source data

**Extended Data Fig. 6:**
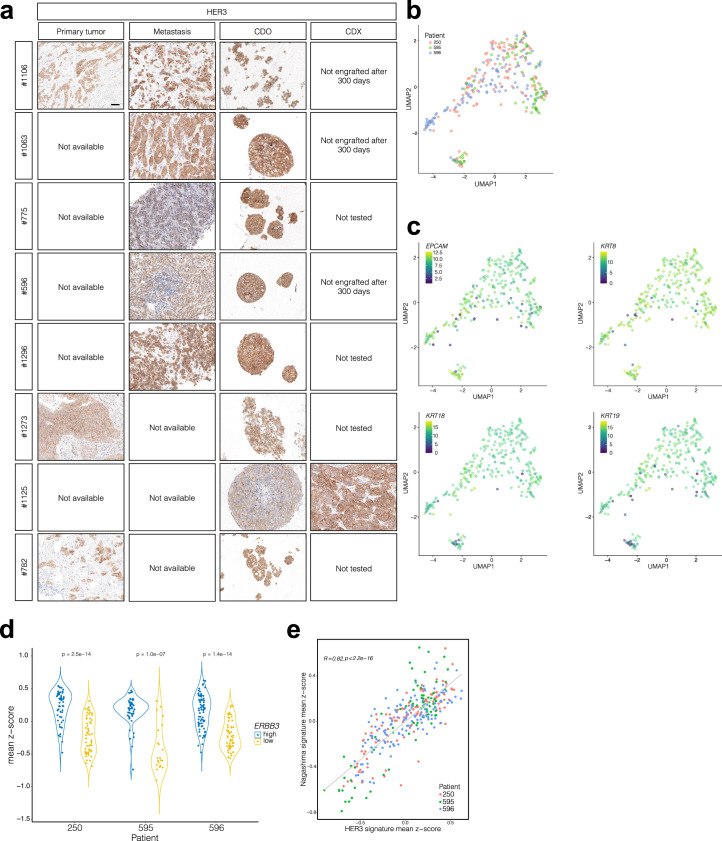
HER3 expression and scRNAseq of CTCs. **a**. Representative IHC picture for HER3 protein expression in patient primary tumor and metastatic lesions (left), *in vitro* CTCs Derived Organoids (CDO) (middle) and xenograft (CDX) (right), n = 1. Scale bar, 50 μm **b**. UMAP plot from scRNAseq analysis. Each dot represents one putative CTC, the color gradient is based on the origin (patient) of the cells. **c**. UMAP plot from scRNAseq analysis. Each dot represents one putative CTC, the color gradient is based on the expression of the indicated gene. **d**. Violin plot representing mean z-scores per cell for *ERBB3*^high^ and *ERBB3*^low^ cell populations. Z-score was computed for each signature gene and the mean of all z-scores was calculated for each cell. Two side t-test. **e**. Scatter plot of mean z-score over HER3-signature genes against mean z-score over NRG1-signature genes (Nagashima^21^). Each point represents a cell colored according to the patient of origin. Coefficient of determination (R^2^) and p-value result from Pearson´s correlation between the NRG1 mean z-scores and the HER3 mean z-scores is reported.

Given the critical role of NRG1 in maintaining CDO survival and proliferation, we next investigated the expression of ERBB family member receptors (EGFR and HER2–HER4) relevant to mediating NRG1 intracellular signaling. Among these, HER3, capable of directly binding NRG1 and preferred dimerization partner of HER2 (ref. ^16^), showed the highest and most consistent, albeit heterogeneous, expression across the different models (Fig. 3a and Extended Data Fig. 5d). HER3 protein expression was detected in CDOs of all subtypes, matched xenograft tumors (CDX) and matched participant primary tumors and metastases (Fig. 3b and Extended Data Fig. 6a).

Single-cell transcriptomic analysis revealed heterogeneous expression of *ERBB3* in three primary uncultured participant CTCs (Fig. 3c,d, Extended Data Fig. 6b,c and Supplementary Table 1). Analysis of differentially expressed genes in *ERBB3*^hi^ compared to *ERBB3*^lo^ clusters yielded 598 differentially regulated genes (Supplementary Table 3) that we used to generate a HER3 gene signature. This signature separated *ERBB3*^hi^ from *ERBB3*^lo^ CTCs within each participant (Extended Data Fig. 6d) and was highly enriched in lung-metastatic cells compared to primary tumor cells in the xenografts (Fig. 3e), indicating that HER3^sign_high^ cells may mediate lung metastasis formation. Importantly, our CTC HER3 signature strongly correlates (*R* = 0.82, *P* < 2.2 × 10^–16^) with a reported functional NRG1 signature (‘Nagashima NRG1 signaling up’^21^), which was obtained after short-term treatment of MCF7 cell lines with NRG1 (Extended Data Fig. 6e). This is consistent with the functional expression of HER3 and activity of its downstream signaling cascade in CTCs of MBCPs.

**Extended Data Fig. 7:**
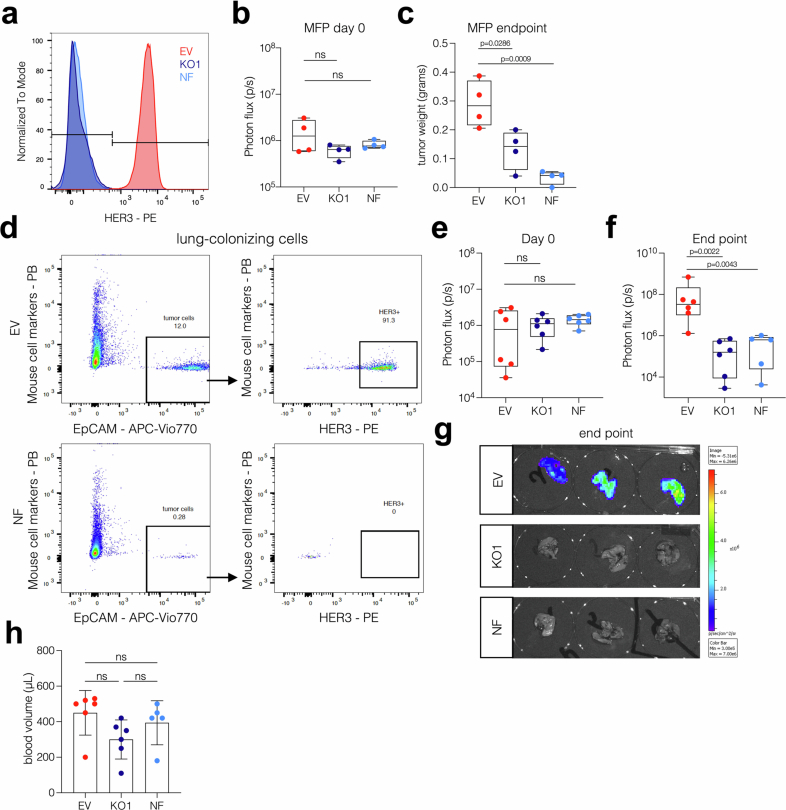
HER3 functional role *in vivo.* **a**. Histogram showing representative flow-cytometry analysis for HER3 expression at the plasma membrane in CTC223 cells transduced with empty vector (EV, red) or two different gRNAs (KO in blue and NF in turquoise), n = 1. Experiments were repeated at least three times independently with similar results. **b**. Boxplot showing the quantification of luminescence signal from transplanted cells (CTC223 cells (EV (red), *ERBB3* KO (blue), and NF (turquoise)) in the mammary fat pad (MFP) of female NSG mice at the day of injection. Each dot represents a mouse, boxs show low and upper quartiles and median line is indicated, whiskers indicate minimum and maximum value, n = 4 mice. Two-tailed Mann Whitney test, ns: not significant vs EV. **c**. Boxplot showing tumor weight in grams at end point. Each dot represents a tumor, n = 4 per condition, boxes show low and upper quartiles and median line is indicated, whiskers indicate minimum and maximum value. Two-tailed Unpaired t test, KO1 vs EV: p = 0.0286, NF vs EV: p = 0.0009. **d**. Representative flow-cytometry plots of lung-colonizing cells at end point. Tumor cells are defined as Blood Lineage^−^ and EpCAM^+^. HER3 expression was checked. **e**. Boxplot showing lung bioluminescence intensity at the day (day 0) of the intravenous (tail vein) injection of CTC223 cells. Each dot represents a mouse, boxs show low and upper quartiles and median line is indicated, whiskers indicate minimum and maximum values, n = 6 mice. Two-tailed Mann Whitney test, ns: not significant vs EV. **f**. Boxplot showing *ex vivo* lung bioluminescence intensity at end point from intravenous injection of CTC223 cells. Each dot represents a mouse, boxs show low and upper quartiles and median line is indicated, whiskers indicate minimum and maximum values, EV, KO1: n = 6, NF: n = 5 mice. Two-tailed Mann Whitney test, KO1 vs EV: p = 0.0022, NF vs EV: p = 0.0043. **g**. Image of *ex vivo* lung bioluminescence from Fig. 3i,j and Extended Data Fig. 7f. **h**. Plot representing the blood volume used to detected CTCs at the end point of the metastatic assay. Each dot represents one mouse, mean ± s.d. id indicated, EV, KO1: n = 6, NF: n = 5 mice. One-way ANOVA, Tukey´s multiple comparisons test, the p value of each comparison is reported. Source data

To functionally dissect the role of HER3, we used CTC223 CDOs, which were established and maintained in G1 medium lacking NRG1 and thus developed independently of exogenous NRG1. We engineered CTC223 cells with an *ERBB3*-knockout (KO) allele or introduced an insertion–deletion-carrying non-functional (NF) allele defective for plasma membrane expression (Extended Data Fig. 7a). As expected, both *ERBB3*-KO cells and *ERBB3*-NF cells completely lacked NRG1-mediated HER3 phosphorylation (Fig. 3f). All edited CDOs showed similar growth kinetics in the absence of NRG1; however, only cells carrying wild-type (WT) *ERBB3* (empty vector (EV)) responded to NRG1 treatment with increased growth, whereas the two mutant CDOs were refractory (Fig. 3g).

**Extended Data Fig. 8:**
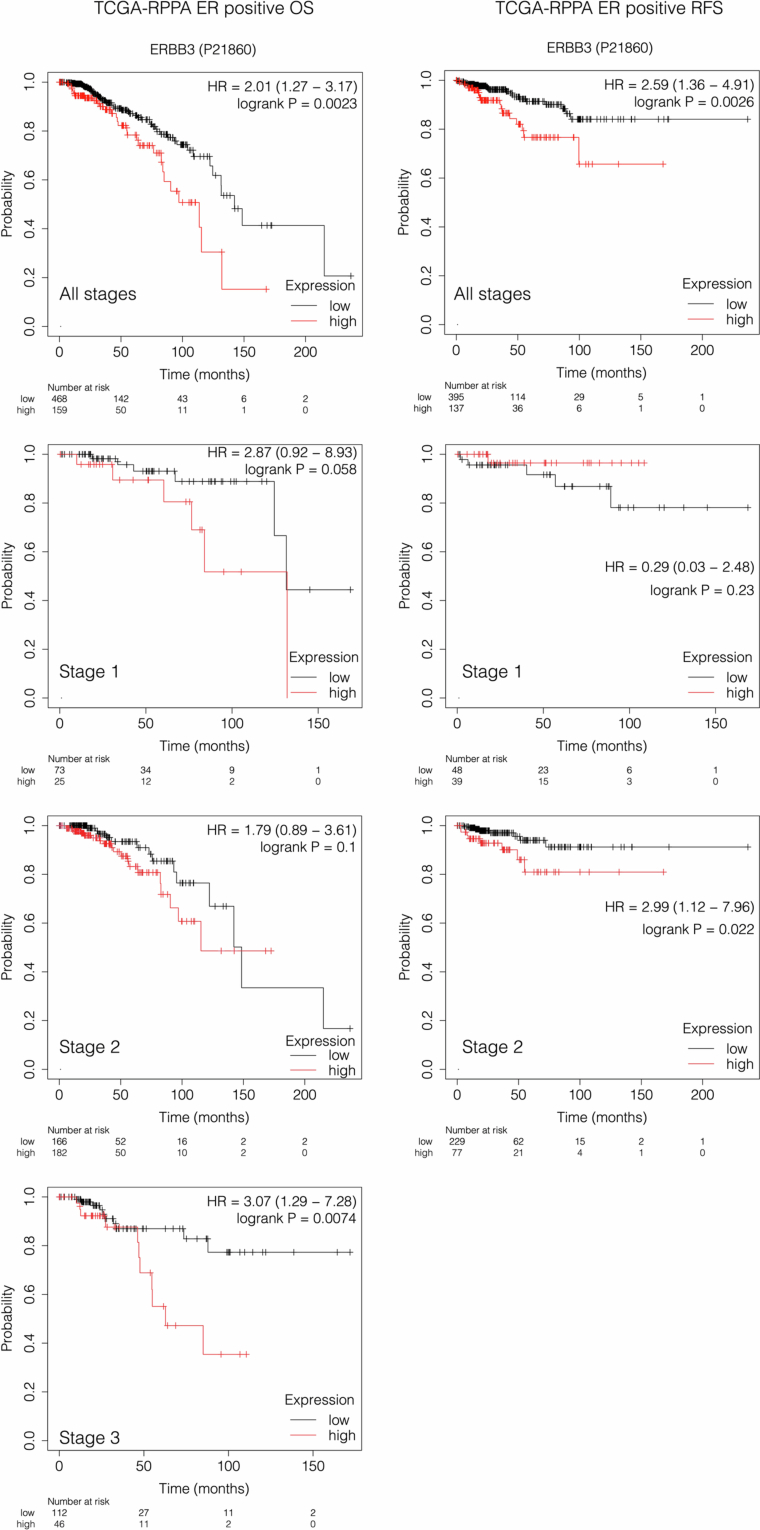
HER3 expression and breast cancer patient survival. Kaplan-Meier curves indicating the overall survival (OS, left panels) and relapse free survival (RFS, right panels) according to HER3 protein expression (low in black and high in red) using the TCGA RPPA cohort filtered for ER positive breast cancer. First row in the plots all stages (1 + 2 + 3) are included, second row only stage 1 is included, third row only stage 2 is included, bottom plot only stage 3 is included.

We next tested whether blocking HER3–NRG1 signaling could affect tumor growth in vivo by transplanting CDOs into the mammary fat pad of NSG mice. *ERBB3*-KO- and *ERBB3*-NF-cell-derived tumors were significantly smaller than *ERBB3*-EV controls (Fig. 3h and Extended Data Fig. 7b,c). To specifically evaluate the role of HER3 in mediating lung colonization independent of primary tumor growth, we performed tail vein injections of each of the three *ERBB3* variant cell lines (Fig. 3i). After 5 months, thousands of *ERBB3*-EV cells (average of 3,430.6; range of 1,649–7,074) colonized the lungs, but none or only a few cells were detected in the *ERBB3*-KO (average of 1.25, range of 0–3) and *ERBB3*-NF groups (average of 22.75, range of 0–71; Fig. 3j, left, and Extended Data Fig. 7d). This was confirmed by bioluminescence quantification (Extended Data Fig. 7e–g). Consistently, we observed an average of 4.4 (range of 0–8.8) CTCs per ml of blood in *ERBB3*-EV transplant recipients, whereas no CTCs were detected in the circulation in the *HER3*-KO or *HER3*-NF cohorts (Fig. 3j, right, and Extended Data Fig. 7h). Importantly, the reintroduction of WT *ERBB3* into *ERBB3*-KO cells (rescue) completely rescued the phenotype, confirming that HER3 is required and sufficient for lung colonization (Fig. 3k). Lastly, interrogation of The Cancer Genome Atlas (TCGA) reverse-phase protein array (RPPA) cohort^29^ revealed that HER3 is associated with poor outcome at the later stage of BC with respect to both relapse-free survival (*P* = 0.0046) and overall survival (*P* = 0.0074; Fig. 3l and Extended Data Fig. 8).

Collectively, these data suggest a crucial role for NRG1–HER3 signaling in CTC dissemination and lung colonization, raising the possibility that high HER3 activity provides CTCs with propagating and lung metastasis-initiating capacity.

### FGFR1 signaling circumvents HER3–NRG1 dependency in CDOs

**Fig. 4:**
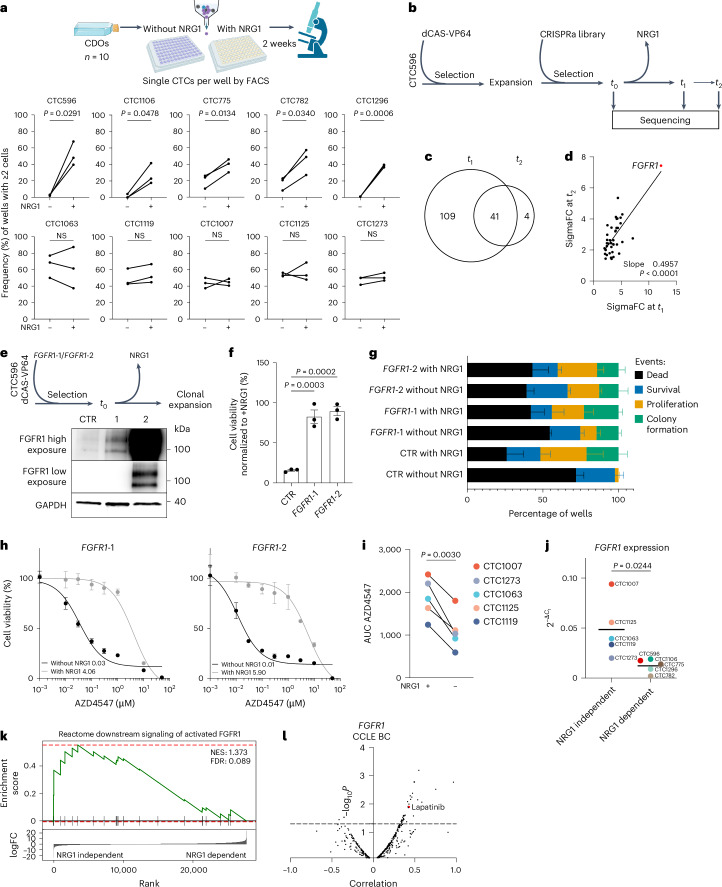
FGFR1 signaling acts as a compensatory pathway and circumvents HER3–NRG1 dependency in CTCs. **a**, Top: clonogenic assay workflow. Bottom: clonogenic assay quantification. Data were analyzed by two-tailed paired *t*-test (CTC596, *P* = 0.0291; CTC1106, *P* = 0.0478; CTC775, *P* = 0.0134; CTC782, *P* = 0.0340; CTC1296, *P* = 0.0006; CTC1063, *P* = 0.6965; CTC1119, *P* = 0.0848; CTC1007, *P* = 0.8611; CTC1125, *P* = 0.7429; CTC1273, *P* = 0.1869 with versus without NRG1; *n* = 3 biological replicates). **b**, CRISPR–dCas9 genome-wide activation screening workflow. **c**, Overlapping enriched genes in *t*_1_ and *t*_2_ compared to *t*_0_ (FDR < 0.05, number of gRNAs ≥ 2). **d**, Correlation plot between SigmaFC at *t*_1_ and *t*_2_ for overlapping genes (*n* = 41). A simple linear regression was calculated, and slope and *P* values are reported. **e**, Top: validation experiment workflow. Bottom: western blot analysis of CTC596 cells overexpressing *FGFR1* (*FGFR1*-1 and *FGFR1*-2) and control (CTR, *n* = 1). **f**, Viability of CTC596 cells expressing dCas9 (CTR) or overexpressing *FGFR1* (*FGFR1*-1 and *FGFR1*-2) without NRG1. Values were normalized to CTR cells with NRG1. Bars represent the mean, error bars indicate s.e.m., and each dot represents an experiment (*n* = 3 biological replicates). Data were analyzed by one-way ANOVA with a Dunnett’s multiple comparisons test (*FGFR1*-1 versus CTR, *P* = 0.0003; *FGFR1*-2 versus CTR, *P* = 0.0002). **g**, Clonogenic assay quantification (*n* = 3 biological replicates). Data were analyzed by two-way ANOVA with a Tukey’s multiple comparisons test. **h**, Dose–response of *FGFR1*-overexpressing cells treated with AZD4547 in the presence or absence of NRG1. Bars represent the mean, and error bars indicate s.e.m.; *n* = 3 biological replicates for all *FGFR1*-1 conditions but NRG1 0.001 µM (*n* = 2), and *n* = 3 for all *FGFR1*-2 conditions but NRG1 0.001 µM (*n* = 2) and NRG1 3 µM (*n* = 1). The average IC_50_ values are reported. **i**, AUC values for the NRG1-independent models in response to treatment with AZD4547 with (left) or without (right) NRG1. Each dot represents the AUC mean derived from two independent experiments. Data were analyzed by two-tailed paired *t*-test; *P* = 0.0030. **j**, *FGFR1* expression in CDOs grouped according to NRG1 dependency. The bars represent the mean. Data were analyzed by two-tailed unpaired *t-*test; *P* = 0.0244. **k**, GSEA using the Reactome downstream signaling of activated FGFR1 signature as the dataset and RNA-seq data from NRG1-dependent and NRG1-independent CDOs as the gene set. **l**, Correlation plot between drug sensitivity and *FGFR1* expression in BC cell lines. Source data

Interestingly, although all established CDOs expressed high levels of HER3, they showed variable dependencies on NRG1 as measured by their clonogenic outgrowth efficiency in the presence or absence of NRG1. We identified an ‘NRG1-dependent’ group, which includes CTC596, CTC1106, CTC782, CTC775 and CTC1296, and an ‘NRG1-independent’ group, which includes CTC1063, CTC1119, CTC1007, CTC1125 and CTC1273 (Fig. 4a).

To identify pathways that can compensate for NRG1–HER3 signaling at the functional level, we performed a genome-wide CRISPR activation (CRISPRa) screen using NRG1-dependent CTC596 CDOs (Fig. 4b). When NRG1 was withdrawn from the medium, we observed an initial massive cell death, followed by regrowth of NRG1-independent clones carrying specific single guide RNA (sgRNA), which we then collected at two different time points (*t*_1_ and *t*_2_). The screen identified 41 genes significantly enriched at both *t*_1_ and *t*_2_ compared to the initial time point (*t*_0_; Fig. 4c and Supplementary Table 4). The top enriched hit was *FGFR1* (Fig. 4d), which is also genetically altered in 20% of MBCs^30^.

**Extended Data Fig. 9:**
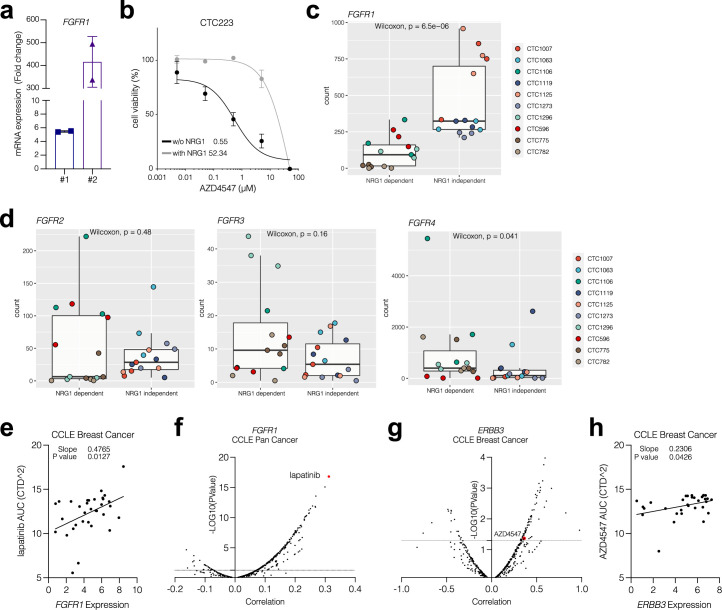
*FGFR1* expression and its correlation with HER3. **a**. Bar plot representing the expression level of *FGFR1* after overexpression in CTC596 using two independent guides (*FGFR1*#1, *FGFR1*#2). Data are shown as mean ± s.d., n = 2 biological replicates. **b**. Plots representing the dose response of CTC223 cells to AZD4547 in the presence (grey) or absence (black) of NRG1. CTB assay was performed after 72 h. The CTB fluorescence value was normalized to the viability of cells without the drug. Bars represent the mean, error bars indicate standard error of the mean, n = 3 biological replicates. The average IC50 values are reported (µM). **c**. Boxplot showing *FGFR1* expression (normalized counts) from RNAseq data. Cells are grouped according to NRG1 dependency (CTC1106, CTC596, CTC1296, CTC782, CTC775 NRG1 dependent models on the left, CTC1007, CTC1125, CTC1063, CTC1119, CTC1273 NRG1 independent models on the right). Boxplots show low and upper quartiles and median line is indicated. Whiskers, 1.5 × interquartile range. Two-sided Wilcoxon test was used. **d**. Boxplots showing *FGFR2*-*3-4* mRNA expression (normalized counts) from RNAseq data. Cells are grouped according to NRG1 dependency (CTC1106, CTC596, CTC1296, CTC782, CTC775 NRG1 dependent models on the left, CTC1007, CTC1125, CTC1063, CTC1119, CTC1273 NRG1 independent models on the right). Boxplots show low and upper quartiles and median line is indicated. Whiskers, 1.5 × interquartile range. Two-sided Wilcoxon test was used. **e**. Correlation plot between Lapatinib sensitivity (AUC) and *FGFR1* expression in n = 33 breast cancer cell lines. Data retrieved from DepMap portal (CTDv2 database). **f**. Correlation plot between drug sensitivity (n = 543 drugs) and *FGFR1* expression in pan-cancer cell lines. Data retrieved from DepMap portal (CTDv2 database). **g**. Correlation plot between drug sensitivity (n = 543 drugs) and *ERBB3* expression in breast cancer cell lines. Data retrieved from DepMap portal (CTDv2 database). **h**. Correlation plot between AZD4547 sensitivity (AUC) and *ERBB3* expression in n = 33 breast cancer cell lines. Data retrieved from DepMap portal (CTDv2 database). Source data

To validate these data, we overexpressed *FGFR1* in CTC596 CDOs using a CRISPRa system with two different sgRNAs (1 and 2), resulting in either 5- or 400-fold *FGFR1* overexpression (Fig. 4e and Extended Data Fig. 9a). Although cell viability of control cells was reduced to 15.6% 72 h after removal of NRG1, as expected, *FGFR1* overexpression was sufficient to rescue this phenotype, achieving 82.66% and 89.48% (*FGFR1* sgRNAs 1 and 2, respectively) of viable cells despite the absence of NRG1 (Fig. 4f). These results were further confirmed by clonogenic assays; although 71.88% of cells died and no colony formation was observed in control cells in the absence of NRG1, *FGFR1*-overexpressing CDOs (sgRNA 1 or sgRNA 2) showed a significantly lower percentage of dead cells (54.51% and 39.23%, respectively) and a higher proportion of proliferating and colony-forming cells in the absence of NRG1 (Fig. 4g). These data suggest that *FGFR1* overexpression fully compensates for the absence of NRG1, leading to a complete rescue of cell viability and clonogenic growth potential.

To functionally validate this finding, we treated *FGFR1*-overexpressing CDOs with a selective FGFR inhibitor (AZD4547)^31^. As shown in Fig. 4h, *FGFR1*-overexpressing CDOs were highly sensitive to AZD4547 in the absence of NRG1. Notably, cell viability reduction induced by FGFR inhibition was completely rescued by adding NRG1. We further validated the mutual compensatory effect of NRG1 and FGFR1 in our NRG1-independent CDX models (Extended Data Fig. 9b) as well as in all five NRG1-independent CDOs (Fig. 4i). Lower sensitivity to AZD4547 was observed in the presence of NRG1, demonstrating that in nongenetically manipulated CDOs, NRG1–HER3 signaling can also rescue cell viability reduction induced by FGFR1 inhibition.

Given the ability of NRG1 to confer resistance to FGFR1 inhibition, we conclude that the FGFR1 and NRG1–HER3 pathways are functionally interdependent in BC cells. This suggests that the cells exhibit adaptive plasticity, enabling them to switch between these pathways to promote survival, proliferation and ultimately metastasis. Accordingly, we investigated whether NRG1-dependent CDOs had lower baseline FGFR1 signaling levels. As shown in Fig. 4j, compared to NRG1-dependent cells, NRG1-independent cells indeed showed overall higher *FGFR1* mRNA expression. These findings were confirmed using RNA-sequencing (RNA-seq) data (Extended Data Fig. 9c) and were specific to *FGFR1* (Extended Data Fig. 9d). Importantly, NRG1-independent CDOs displayed a corresponding enrichment in an FGFR1 activation signature (Fig. 4k). To corroborate these findings in a larger and independent cohort, we interrogated DepMap datasets. A positive correlation between *FGFR1* expression and resistance to lapatinib was observed not only in BC cell lines (Fig. 4l and Extended Data Fig. 9e) but also for the entire PanCancer dataset (Extended Data Fig. 9f). In line with this, *ERBB3* expression positively correlated with resistance to the FGFR inhibitor AZD4547 (Extended Data Fig. 9g,h).

Together, these results demonstrate that the plastic engagement of either FGFR1 or HER3 allows cancer cells to adapt to different environmental cues.

### Combined NRG1 and FGF inhibition eradicates CDOs

**Fig. 5:**
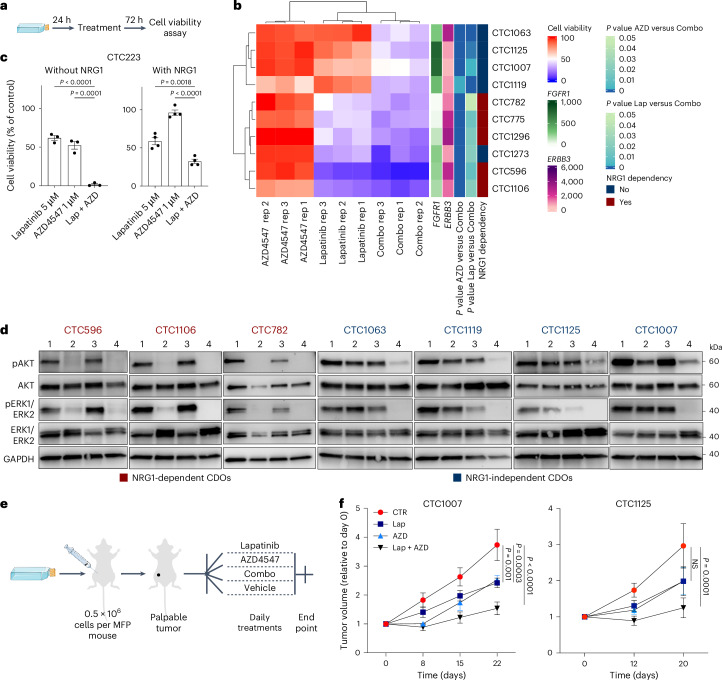
Combined inhibition of NRG1 and FGF signaling leads to CDOs elimination. **a**, Experimental design of the drug screening experiment in CDOs. **b**, Heat map showing relative viability of different CDOs (rows) in the presence of lapatinib (Lap), AZD4547 (AZD) or lapatinb + AZD4547 (combo). Each column represents one biological replicate; each condition has three biological replicates (rep1–3). *FGFR1* and *ERBB3* expression (RNA-seq normalized counts) as well as statistical analyses (one-way ANOVA with a Dunnett’s multiple comparisons test) were added as additional annotation. A CTB assay was performed after 72 h of treatment. CTB fluorescence values were normalized to the viability of control cells incubated with vehicle only. **c**, Bar plot showing relative viability of CTC223 cells in response to lapatinib or AZD4547 alone or lapatinb + AZD4547 in the absence (left, *n* = 3 biological replicates) or presence (right, *n* = 4 biological replicates) of NRG1. A CTB assay was performed 72 h after treatment. CTB fluorescence values were normalized to the viability of control cells incubated with vehicle only. Bars represent the mean, error bars indicate s.e.m., and each dot represents an experiment. Data were analyzed by one-way ANOVA with a Tukey’s multiple comparisons test (Lap + AZD versus Lap, *P* < 0.0001; Lap + AZD versus AZD, *P* = 0.0001 without NRG1; Lap + AZD versus Lap, *P* = 0.0018; Lap + AZD versus AZD, *P* < 0.0001 with NRG1). **d**, Western blot analysis of whole-cell protein lysates derived from CDOs treated with DMSO, lapatinib, AZD4547 or lapatinb + AZD4547 for 12 h. Phosphorylated and total protein kinase B (AKT) and ERK1/ERK2 were detected. GAPDH was used as the loading control; *n* = 1; lane 1, DMSO; lane 2, 5 μM lapatinb; lane 3, 1 μM AZD4547; lane 4, lapatinb + AZD4547. **e**, Experimental design of drug treatment in vivo. **f**, Plots showing increases in tumor volume over time in PDXs treated with vehicle (red), lapatinib (blue), AZD4547 (turquoise) or lapatinib + AZD4547 (black). Tumor volume was measured with a digital caliper and normalized to the volume at baseline (day 0). Data are shown as mean ± s.e.m.; *n* = 6 mice per group. Data were analyzed by two-way ANOVA with a Tukey’s multiple comparisons test (CTC1007 Lap + AZD versus CTR, *P* < 0.0001; Lap versus CTR, *P* = 0.0003; AZD versus CTR, *P* = 0.001; CTC1125 Lap + AZD versus CTR, *P* = 0.0001; Lap versus CTR and AZD versus CTR, not significant). Source data

Driven by the observation of the mutual plasticity between NRG1–HER3 and FGFR1 signaling, we hypothesized that concurrent inhibition of both pathways would be most effective in eliminating CDOs. We tested this hypothesis by performing viability assays on both NRG1-dependent and NRG1-independent CDOs treated with lapatinib or AZD4547 alone or in combination (Fig. 5a). Although NRG1-dependent CDOs (CTC596, CTC1106, CTC782, CTC775 and CTC1296) were highly sensitive to lapatinib, NRG1-independent CDOs were more resistant (Fig. 5b). Generally, CDOs showed resistance to AZD4546 in the presence of NRG1. As expected, combination treatment with lapatinib and AZD4547 was significantly more effective than either of the two drugs alone in all CDOs, regardless of their dependency on NRG1. These findings were also confirmed in the NRG1-independent CDX model. Although combination treatments in the absence of NRG1 eliminated all viable cells, NRG1 stimulation supported cellular viability in the presence of both drugs yet significantly less than treatment with either of the drugs alone (Fig. 5c). Furthermore, although lapatinib alone caused strong suppression of AKT/ERK activity downstream of HER2/HER3 stimulation in NRG1-dependent lines, combination treatment blocked AKT/ERK phosphorylation in both NRG1-dependent and NRG1-independent cells (Fig. 5d).

As proof of concept, with the aim of translating these findings into an in vivo setting, we transplanted two TNBC NRG1-independent CDOs (CTC1007 and CTC1125) into the mammary fat pads of NSG mice and performed in vivo drug treatment (Fig. 5e). As shown in Fig. 5f, although a mild tumor reduction was observed when mice were treated with either lapatinib or AZD4547 alone, a stronger reduction was achieved following combined treatment in both models.

In conclusion, we demonstrate that simultaneous inhibition of NRG1–HER3 and FGF–FGR1 signaling pathways impaired CTC survival and proliferation in vitro and tumor formation in vivo.

### *FGFR1* expression in therapy-resistant BC cells

**Fig. 6:**
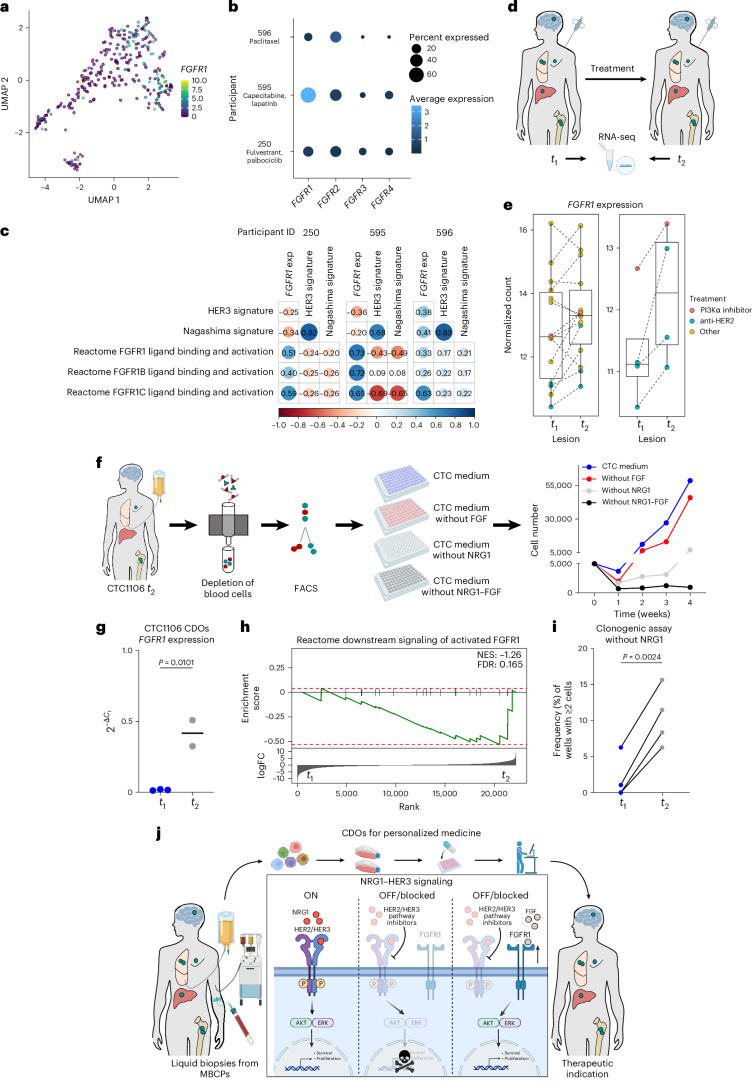
*FGFR1* expression is functionally relevant in therapy-resistant BC cells. **a**, Uniform manifold approximation and projection (UMAP) plot from scRNA-seq analysis from Fig. 3c. Each dot represents one putative CTC, and the color gradient is based on *FGFR1* expression. **b**, Dot plot showing *FGFR1*, *FGFR2*, *FGFR3* and *FGFR4* expression in putative CTCs from each participant (CTC250, CTC595 and CTC596). Below each participant ID, the treatment received before collection of the liquid biopsy is specified (more details are available in Supplementary Table 1). **c**, Correlation dot plot of HER3, NRG1 and FGFR1 signatures. Mean *z* scores per CTC were computed for the HER3 signature, the Nagashima NRG1 signature and three FGFR1-related Reactome gene sets and were correlated against *FGFR1* expression (exp) and the HER3 and Nagashima signature scores. Pearson correlations were computed per participant using only the non-*ERBB3*-expressing CTCs. **d**, Schematic workflow of longitudinal sample collection in the CATCH cohort for transcriptomic analysis. **e**, Left: box plot showing *FGFR1* expression levels assessed by RNA-seq in matched biopsies before and after drug treatment. Treatments are indicated by color. Righ:, box plot showing *FGFR1* expression in matched biopsies before and after drug treatment with either PI3Kα inhibitors (pink) or anti-HER2 (turquoise). Each dot represents a participant. **f**, Growth curve of CTC1106 cells (effusion, *t*_2_) in the reported medium. Cells were counted weekly for 1 month. Dots represent the mean; *n* = 3 technical replicates. **g**, Plot representing *FGFR1* expression in CTC1106 CDOs established from the first (*t*_1_, blue; *n* = 3 biological replicates) and second (*t*_2_, gray; *n* = 2 biological replicates) time points measured. Data were analyzed by two-tailed unpaired *t-*test; *P* = 0.0101. **h**, GSEA using the Reactome downstream signaling of activated FGFR1 signature on data from CTC1106 CDOs established from the first (*t*_1_) and second (*t*_2_) time points. **i**, Plot showing the frequency of wells with two or more live cells in a clonogenic assay using CTC1106 models. Counting was performed 14 days after single-cell FACS seeding in CTC medium without NRG1. Data were analyzed by two-tailed paired *t*-test; *P* = 0.0024 (*n* = 4 biological replicates). **j**, Graphical abstract. Source data

**Extended Data Fig. 10:**
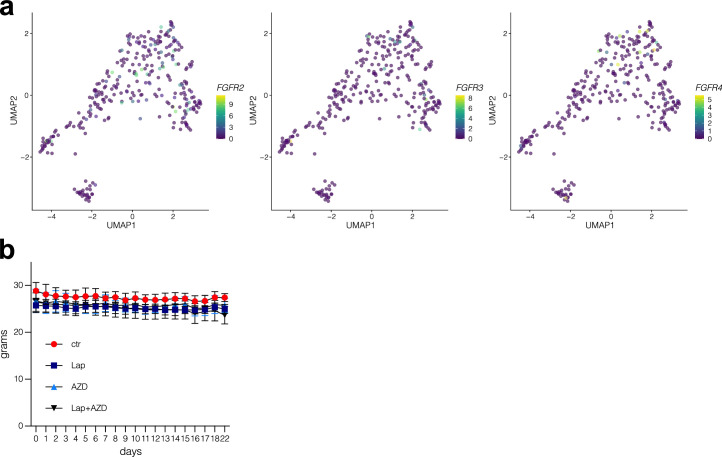
*FGFRs* expression in CTCs and body weight during in vivo treatments. **a**. UMAP plot from scRNAseq analysis. Each dot represents one putative CTC, the color gradient is based on the expression of the indicated gene. **b**. Plot showing body weights of mice monitored over time in PDX1007 treated with vehicle (red), Lapatinib (blue), AZD4547 (turquoise) or Lapatinib+AZD4547 (black). Data are mean ± standard deviation, n = 6 mice per group. Source data

Considering that, after initial response, most individuals will ultimately develop resistance to HER2-targeted therapies^32^, we first interrogated our single-cell RNA-seq (scRNA-seq) dataset to explore *FGFR* gene expression in uncultured MBCP-derived CTCs (Fig. 6a and Extended Data Fig. 10a). *FGFR1* expression levels were heterogeneous and higher in participants treated with anti-HER2/HER3 therapy regimens administered before liquid biopsy collection (Fig. 6b (participant CTC595 progressed on different lines of anti-HER2/HER3 therapy) and Supplementary Table 1). *ERBB3*^–^ CTCs showed a positive correlation with *FGFR1* expression and functional signatures representing FGFR1 activation in all three participants (*R* = 0.26–0.73). Interestingly, the strongest correlations (*R* = 0.65–0.73) were found in participant 595 (Fig. 6c). These analyses suggest the functional activity of FGFR1 and its downstream pathway in *ERBB3*^–^ CTCs, particularly in individuals resistant to HER2/HER3-targeted therapy.

To test whether individuals who developed resistance to HER2/HER3 or PI3K inhibitors (HER2/HER3 downstream effector) show increased *FGFR1* expression, we collected RNA-seq data from the Heidelberg CATCH cohort^25^ of matched longitudinal biopsies before (*t*_1_) and after (*t*_2_; progressive disease) therapy (Fig. 6d). Heterogeneous *FGFR1* expression was observed in all (*n* = 14) matched longitudinal samples (Fig. 6e). However, in individuals receiving HER2/HER3 or PI3KA inhibitors (*n* = 4), increased *FGFR1* expression was observed at *t*_2_. This observation supports the finding that FGFR1 pathway activation represents an escape mechanism in individuals treated with HER2/HER3 signaling pathway-inhibiting drugs.

To further validate the role of the NRG1–HER3 and FGF–FGFR1 pathways in CTC survival and proliferation, we processed a liquid biopsy from participant CTC1106 (*t*_2_; Fig. 2i). Following hematopoietic cell depletion and live-cell sorting via FACS, the isolated CTCs were plated in complete CTC medium but with the following modifications: (1) standard with NRG1 and FGF, (2) without FGF, (3) without NRG1 and (4) without both NRG1 and FGF. As expected, complete CTC medium supported initial CTC survival and subsequent proliferation over time. Conversely, the absence of both NRG1 and FGF in the CTC medium resulted in a dramatic reduction of CTC survival and failed to sustain their proliferation, demonstrating that both NRG1 and FGF are essential. In medium lacking only FGF, CTC growth was modestly impaired. However, culturing in the absence of NRG1 (gray line) resulted in substantially reduced CTC expansion over time, highlighting its critical role in CTC proliferation and survival (Fig. 6f).

Finally, using the longitudinal CTC1106 CDOs (Fig. 2i), we further assessed the plastic interplay between FGFR1 and HER3. Notably, *FGFR1* expression was significantly increased after alpelisib treatment (*t*_2_; Fig. 6g), which correlated with an enrichment of the FGFR1 activation signature (Fig. 6h). We then functionally linked the increased expression of *FGFR1* with a reduction of NRG1 dependency. As shown in Fig. 6i, CTC1106 *t*_2_ showed a significantly stronger clonogenic ability in the absence of NRG1 than CTC1106 *t*_1_. These data show the relevance of the NRG1–HER3 and FGF–FGFR1 compensatory mechanism in longitudinal samples, suggesting *FGFR1* upregulation as a potential escape mechanism of BC cells that develop treatment resistance (Fig. 6j).

## Discussion

To date, MBC is an incurable disease and represents the second leading cause of death from cancer among women^33^. CTCs are the source of metastasis, but long-term expansion in vitro has been a major challenge preventing their functional interrogation. Here, we report the establishment and characterization of 17 long-term CDOs originating from liquid biopsies derived from 16 MBCPs spanning the different BC subtypes. Although 6 models were obtained from CDXs or EDXs, 11 CDOs were generated directly from effusion samples or peripheral blood (either by DLA or blood withdrawal) without any initial pre-expansion in vivo. Importantly, these CDOs can allow the investigation of molecular pathways crucial for CTC survival and can also be used as a preclinical model suitable for drug screening and therapy resistance studies in a personalized approach. Moreover, our platform is suitable for serial longitudinal isolation and expansion of tumor cells from MBCPs to monitor disease progression and therapy responses. Although our cohort size is limited and the paucity of data available from BC CTC cultures hinders statistically significant conclusions, copy number and single-nucleotide variant analyses of the CDOs do not provide evidence that our culture condition is selective for the growth of CTCs from individuals with specific genomic profiles. The most critical variable appears to be the starting number of cultured CTCs. By focusing on individuals with over 100 CTCs, this study necessarily limited its applicability to a narrower range of individuals. However, we showed that the use of alternative liquid biopsies like DLA or pleural and peritoneal effusions can overcome this limitation.

CTCs represent a heterogeneous population, with only a fraction able to survive, extravasate and seed distant metastases. The ability of certain CTCs to survive is a crucial step during metastasis formation. Our data identify NRG1–HER3 signaling as one of the key players mediating this process. Indeed, NRG1 enabled the establishment of robust in vitro conditions, which allowed initiation and long-term expansion of primary CTCs. Accordingly, in vivo data show that HER3^+^ CTCs represent a highly aggressive subpopulation with superior survival and metastatic potential.

In response to extrinsic factors, such as drug treatments, phenotypic plasticity can contribute to the heterogeneity of CTCs, leading to treatment resistance^11^. Here, we provide evidence of a complementary mechanism of action in which cells can switch between HER3 and FGFR1 to promote growth and survival via AKT–ERK pathways. As a consequence, combined blockade of NRG1 and FGF signaling resulted in sustained growth inhibition of CDOs both in vitro and in vivo. These findings are in line with other studies where the cross-talk between FGFRs and ERBB family members have been hypothesized to mediate therapy resistance in liver cancer^34,35^, lung cancer^36^ and BC^37^. In addition, amplification of FGF signaling has been shown to promote resistance to HER2 inhibition in HER2^+^ BC^38^. The ability of FGFR1 to upregulate *NRG1* was recently reported in pancreatic ductal adenocarcinoma^39^; however, we did not observe this phenomenon in our models.

To inhibit the HER2/HER3 pathway, we used lapatinib, a reversible, ATP-competitive tyrosine kinase inhibitor of HER2 that has been approved for treatment of HER2^+^ BC for a decade. Alternative strategies are at different stages of approval and may offer more effective options. Based on our in vitro, in vivo and ex vivo data, MBCPs with advanced disease and HER3-expressing CTCs will likely benefit from treatment with last-generation HER3 inhibitors, such as patritumab deruxtecan, a HER3-directed antibody-drug conjugate recently approved for the treatment of metastatic *EGFR*-mutated non-small cell lung cancer^40,41^ with encouraging results in early BC^42^. Ideally, the subsequent addition of an FGFR1 inhibitor would prevent potential adaptive resistance mediated by cell plasticity.

## Methods

All research performed in this study complied with all relevant German ethical regulations.

### Clinical specimens

Liquid biopsy samples (pleural and ascitic effusions and peripheral blood withdrawals) were obtained from MBCPs participating in the CATCH (Comprehensive Assessment of Clinical Features and Biomarkers to Identify Patients with Advanced or Metastatic Breast Cancer for Marker Driven Trials in Humans) trial at the Division of Gynecologic Oncology, NCT Heidelberg (case number S-164/2017). Written informed consent was obtained from all participants.

Participant and tumor characteristics are summarized in Supplementary Table 1. CTC-specific assessments were further approved by the ethical committee of the University of Heidelberg (case number S295/2009) and University of Mannheim (2010-024238-46).

### EDX and CDX models

The procedure for generating orthotopic xenografts from effusion was first described by Al-Hajj et al.^43^ and was recently refined by our group^19^; peripheral blood withdrawal was previously described by our group^1^.

### CTC enumeration

Blood samples were collected in CellSave tubes (Menarini Silicon Biosystems) and run with CellSearch (Menarini) using the CTC kit (Menarini) at the Department of Tumor Biology, Hamburg, according to the manufacturer’s guidelines.

### Blood samples

Peripheral blood samples were directly collected in Vacutainer CPT tubes (BD Biosciences) and processed following the manufacturer’s guidelines.

### Leukapheresates

MBCPs with ≥10 CTCs per 7.5 ml of blood were asked to participate in the CTC leukapheresis study, approved by the ethical committee of the University of Heidelberg (case number S-408/2013). DLA was performed at the Medical University Hospital Heidelberg using a Spectra Optia (Terumo BCT) according to manufacturer’s instructions. Plasma and CTCs were collected using the cMNC program with a reduced packing factor of 4.5 and with 2–3% hematocrit.

Apheresate products were immediately collected and processed under sterile conditions. Depletion of blood cells was performed using Miltenyi Biotec microbeads (anti-CD45, 130-045-801; anti-CD3, 130-050-101; anti-CD31, 130-091-935; anti-CD16, 130-045-701; anti-CD235a, 130-050-501; each 20 µl per 10^7^ cells).

### FACS and flow cytometry

Cells were stained in PBS + 1% bovine serum albumin (BSA) and 2 mM EDTA using the following antibodies: EpCAM-FITC and APC-Vio770 (clone HEA-125, REA764 Miltenyi Biotec, 1:50), CD45-VioBlue (REA747 clone, Miltenyi Biotec, 1:50), HER3-PE (66223 clone, FAB3481P, R&D, 1:20), CD31-VioBlue (clone AC128, Miltenyi Biotec, 1:50), CD16-VioBlue (clone REA423, Miltenyi Biotec, 1:50), CD41-VioBlue (clone REA386, Miltenyi Biotec, 1:50) and CD235a-VioBlue (clone REA175, Miltenyi Biotec, 1:50). Propidium iodide (PI; P3566, Thermo Fisher Scientific, 1:1,000) or DAPI (D1306, Thermo Fisher Scientific, 1:1,000) was used to exclude dead cells. The following antibodies were used for the analysis of xenograft-derived samples (all anti-mouse, PacificBlue, Biolegend): CD45 clone 30-F11 (103116, 1:1,000), CD11b clone M1/70 (101226, 1:2,000), TER-119/Ly-76 clone TER-119 (116223, 1:200), Ly-6G clone 1A8 (A25985, 1:2,000), CD31 clone 390 (102422, 1:1,000) and H-2K^d^ clone SF1-1.1 (116629, 1:50).

Cells were sorted and analyzed using a FACSAria Fusion and LSR Fortessa (BD Biosciences). BD FACSDiva software and FlowJo software were used for analysis.

### Determination of NRG1 blood concentration

NRG1 concentration in blood samples from MBCPs was measured with a Human NRG1 ELISA kit (Thermo Fisher Scientific) according to the manufacturer’s instructions. Absorbance was determined on a SpectraMax iD3 microplate reader.

### Long-term in vitro expansion of CTCs

Immediately after FACS or immunomagnetic sorting, the CTC-enriched cell suspension was centrifuged, the supernatant was carefully removed, and the pellet was resuspended in the appropriate volume of CTC medium, transferred to a plate (Corning, Primaria) and cultured at 37 °C with 5% CO_2_ and 5% O_2_. Medium was partially replaced every 48–72 h. When cluster formation was detected, cells were collected, dissociated with Accutase (Sigma, Life Technologies) and expanded in larger plates and subsequently in flasks. Floating three-dimensional collagen gels with a final concentration of 1.3 mg ml^–1^ collagen I (Corning) were prepared as previously described with minor modifications^20^. NRG1 concentration used in the medium and in all experiments was 20 ng ml^–1^ unless otherwise specified. The medium recipe was licensed to Miltenyi Biotec, and the optimized formulation is available under the name ‘Breast TumorMACS Medium’; only Y27632 has to be added separately according to the manufacturer’s instructions (StemMACS Y27632, 130-103-922).

The cells were confirmed to be negative for contaminants (no *Mycoplasma*, Squirrel Monkey Retrovirus, Epstein-Barr virus or interspecies contamination was detected) by the Multiplex Cell Contamination Test (Multiplexion)^44^. To test cross-contamination with commercially available cell lines, single-nucleotide polymorphism fingerprints were extracted from RNA-seq data on CDOs in vitro and publicly available RNA-seq data from BC and acute myeloid leukemia cell lines from CCLE^45^ (PRJNA523380) using ExtractFingerprints and CrosscheckFingerprints from GATK4 (ref. ^46^). Briefly, given a BAM/SAM/VCF file, single-nucleotide polymorphisms in specific highly variable genomic locations were extracted and calculated. Concordance between fingerprints was estimated, and the logarithm of the odds score for identity was calculated (Extended Data Fig. 4a).

The match between CDOs and corresponding participant material was confirmed by ExtractFingerprints and CrosscheckFingerprints from GATK4 on whole-genome sequencing data (Extended Data Fig. 4b).

### Clonogenic assay

Single-cell suspensions were obtained by treating cells with Accutase at 37 °C for 5 min. Cells were washed with PBS, spun down, resuspended in a PBS solution containing 1% BSA, 2 mM EDTA and DAPI and filtered through a 40-µm cell strainer. Cells were sorted using a FACSAria Fusion (BD Biosciences) coupled with BD FACSDiva Software using a 100-μm nozzle. After morphological gating using forward and side scatter, we excluded duplets and dead cells (DAPI^+^) and sorted one single cell per each well of a 96-well plate, previously filled with 200 µl of medium. All plates were maintained at 37 °C with 5% CO_2_ and 20.9% O_2_ for normoxia and 5% O_2_ for hypoxia. After 2 or 4 weeks, cells were checked and counted under brightfield microscopy.

### CRISPR–Cas9-mediated *ERBB3* KO

*ERBB3*-KO gRNA sequences (Supplementary Table 1) were cloned into the pLKO.005 backbone of the pLKO5.sgRNA.EFS.tRFP657 plasmid, as previously described^47,48^. The correct assembly of the resulting DNA plasmids was confirmed using Sanger sequencing. Lentivirus production was performed using a second-generation lentivirus system (psPAX2, Addgene plasmid 12260; pMD2.G, Addgene plasmid 12259). Viral supernatant was collected 48 h after transfection, passed through a 450-nm filter, ultracentrifuged at 4 °C for 120 min, resuspended in cold PBS and stored at −80 °C until use. CTC223 cells were transduced with pCW-Cas9-tGFP plasmid. After 4 days of incubation, the GFP–Cas9^+^ CTC223 cells were sorted by FACS, expanded and transduced with pLK05-gRNA1-tRFP (*ERBB3*-KO1), pLK05-gRNA2-tRFP (*ERBB3*-NF) or pLK05-EV-tRFP (EV). GFP^+^tRFP^+^ cells were sorted by FACS and expanded. After 10 days, *ERBB3* KO was induced by adding 1 μM doxycycline hydrochloride to the medium for 96 h and exchanging the medium after 48 h.

### Cell viability assay

Cell viability was measured using the CTB (Promega) assay. Specifically, 8,000–10,000 cells were seeded under the appropriate medium conditions in each well of a 96-well multiwell plate. The day after, the following compounds were added: AZD4547 (S2801), lapatinib (S2111), taselisib (S7103) and alpelisib (S2814; all purchased from Selleckchem).

### Cell cycle analysis

In total, 1 × 10^5^ cells per well were seeded in G1 medium. After 24 h, the medium was replaced with G1, G2, G1 + NRG1, G1 + Y27632 and G1 + NRG1 + Y27632. After 48 h, 5-ethynyl-2′-deoxyuridine (EdU; 1:1,000) of a Click-iT Plus EdU Alexa Fluor 647 Flow Cytometry Assay kit (Invitrogen) was added. After 3 h, cells were processed according to the manufacturer’s instructions and analyzed using an LSR Fortessa.

### Apoptosis assay

In total, 1 × 10^5^ cells per well were seeded in G1, G2, G2 without NRG1 or G2 without Y27632. After 48 or 120 h, cells were washed with PBS and detached with Accutase. Cells were then stained in 1× Annexin V Binding buffer with 1:20 PI and 1:100 Annexin V-PE. After 15 min of incubation at room temperature, samples were measured using an LSR Fortessa. The fractions of alive (PI^–^Annexin^–^), early apoptotic (PI^–^Annexin^+^), late apoptotic (PI^+^Annexin^+^) and necrotic (PI^+^Annexin^–^) cells were determined using FlowJo software.

### Synergism between NRG1 and Y27632

To investigate the synergistic effect of NRG1 and Y27632 on CTC proliferation, we used a linear model to describe cell viability. This model included binary variables for NRG1 treatment, Y27632 treatment and their combination. To test for synergy, we compared two generalized linear mixed models: one with and one without an interaction term for the double treatment. An ANOVA revealed a significant improvement in the model with the interaction term (*P* = 1.25 × 10^–6^), indicating a synergistic effect.

### Mouse studies

Animal care and procedures followed German legal regulations and were previously approved by the governmental review board of the state of Baden-Württemberg, operated by the local Animal Welfare Office (Regierungspräsidium Karlsruhe) under license numbers G-240/11, G-115/17 and G-104/22. Mice were housed in individually ventilated cages under temperature and humidity control. Cages contained an enriched environment with bedding material.

#### In vivo preclinical model for micrometastases

Luciferase-labeled CDX models were injected into the fourth mammary fat pad of female NSG mice that were at least 6 weeks old. Cells were resuspended in a 1:1 ratio of sterile PBS and growth factor-reduced Matrigel (BD). All mice received subcutaneous implantation of β-estradiol as solid pellets (Innovative Research of America), as previously described^1^. Primary tumors were resected after reaching a size of ~0.5 cm^3^. Micromestastasis formation was monitored by measuring bioluminescent signal using an IVIS Spectrum Xenogen device (Caliper Life Sciences). Animals were killed, and micrometastasis-containing organs were collected.

#### CDX generation

NSG mice were transplanted with 1 × 10^6^ cells in the fourth mammary fat pad, as described above. Tumor growth was monitored, and mice were killed when end point criteria were reached.

#### In vivo lung colonization assay

In total, 1 × 10^5^ cells in 100 µl of PBS were injected via the tail vein. Lung colonization was monitored by measuring bioluminescent signal using an IVIS Spectrum Xenogen machine (Caliper Life Sciences). Bioluminescence analysis was performed using Living Image software version 4.4 (Caliper Life Sciences).

#### In vivo drug treatments

Tumor growth was monitored, and drug treatment was initiated when primary tumors were palpable. Mice were treated by oral gavage daily with vehicle (0.1% Tween 80/0.5% Na-CMC, 100 μl), 100 mg per kg (body weight) lapatinib, 10 mg per kg (body weight) AZD4547 or 100 mg per kg (body weight) lapatinib + 10 mg per kg (body weight) AZD4547 all in 0.1% Tween 80/0.5% Na-CMC (100 µl). Tumor size was recorded weekly using digital calipers. Animal weight was recorded daily to monitor potential drug toxicity. At the experimental end point, the maximal tumor size (1.5 cm^3^) was not exceeded. None of the treatments had major toxic effects in vivo (Extended Data Fig. 10b).

### Gene expression analysis by quantitative PCR

Cells were collected, washed with PBS and centrifuged. RNA extraction was performed using miRNeasy Mini kit (Qiagen) or PicoPure kit (Thermo Fisher), depending on the cell number, following the manufacturers’ instructions. RNA concentration and purity were determined using a NanoDrop spectrophotometer. Reverse transcription (RT) was performed using a High-Capacity cDNA Reverse Transcription kit (Applied Biosciences) following the manufacturer’s guidelines. Quantitative PCR was performed using 0.5 μl of forward and reverse primers (10 μM stock solution), 5 μl of Power SYBR Green PCR master mix (Life Technologies), 3 μl of nuclease-free water and 1 μl of 1:10 diluted cDNA. The reaction was run on a Thermo Fisher ViiA-7 Real-time PCR system. Data were analyzed using the comparative cycling threshold (ΔΔ*C*_t_) method. Primers are listed in Supplementary Table 5.

### Gene expression analysis of the PDX model

Primary tumor and metastatic tissues derived from our PDX mouse model were freshly processed using the GentleMACS system (Miltenyi Biotec) to obtain a single-cell suspension. Live tumor cells were sorted by FACS into RNA lysis buffer (Arcturus PicoPure RNA Isolation kit, Life Technologies, Invitrogen). RNA was isolated according to the manufacturer’s instructions. Gene expression analysis was performed using Affy Human U133Plus 2.0 at the Genomics and Proteomics Core Facility of the German Cancer Research Center (GPCF DKFZ, Heidelberg).

To test differences in lung metastases versus primary tumors, genes were ranked based on log fold change. GSEA was performed with the R package gage (v2.36.0) and default settings using the C2 curated gene sets from MSigDB (v7.4). For the top hit (Nagashima NRG1 signaling up) as well as our custom HER3 signature (Supplementary Table 3 and scRNA-seq analysis), GSEA was performed with the R package fgsea (v1.12.0) and default settings to produce the plots in Figs. 1d and 3e, respectively.

### Western blotting

Cells were lysed in RIPA-based protein lysis buffer. Protein concentration was determined using a Pierce BCA Protein Assay kit (Thermo). After blocking for 1 h at room temperature with 1× Tris-buffered saline and Tween 20 buffer containing 5% BSA, membranes were incubated overnight with the appropriate primary antibodies diluted 1:1,000 in blocking buffer. Antibodies to phospho-HER3/ERBB3 (Tyr 1289; 21D3; rabbit monoclonal 4791), HER3/ERBB3 (D22C5; XP rabbit monoclonal 12708), FGFR1 (D8E4; XP rabbit monoclonal 9740), phospho-AKT (Ser 473; D9E; XP rabbit monoclonal 4060), AKT (pan; C67E7; rabbit monoclonal 4691), phospho-p44/42 MAPK (ERK1/ERK2; Thr 202/Tyr 204; D13.14.4E; XP rabbit monoclonal 4370), p44/42 MAPK (ERK1/ERK2; 137F5; rabbit monoclonal 4695), phospho-FAK (Tyr 397; D20B1; rabbit monoclonal 8556), FAK (rabbit polyclonal 3285) and GAPDH (14C10; rabbit monoclonal 2118) were purchased from Cell Signaling Technology. Monoclonal anti-α-tubulin T5168 was purchased from Sigma-Aldrich.

### IHC analysis

Sections from fixed CDOs or tissues were obtained, processed and stained as previously described^49^, with minor modifications. The following antibodies were used: anti-EpCAM (Agilent Dako, clone Ber-EP4, 1:100), anti-human KRT19 (Agilent Dako, clone RCK108, 1:50), anti-human Ki-67 (Agilent Dako, clone Ki-67, 1:1,000), anti-ERα (Thermo Fisher Scientific, clone SP1, 1:50), anti-CDH1 (Agilent Dako, clone M3612, 1:30) and anti-vimentin (Agilent Dako, clone M7020, 1:1,000). For HER3 expression, we incubated samples with anti-HER3/ERBB3 (D22C5, XP rabbit monoclonal 12708, 1:50) at 4 °C overnight and used heat-induced antigen unmasking with damp heat at 90 °C with EDTA unmasking solution (pH 9 (1:10); 14747 Signal Stain) for 30 min. Sections were scanned using a Zeiss AxioScan, and representative images are shown.

### Targeted next-generation sequencing of somatic mutations

Genomic DNA was extracted from CDOs and CDXs. DNA concentration was assessed by fluorimetric measurement using a QuBit 3.0, and the amount of amplifiable DNA (sequencing-grade quality) was determined using a quantitative assay (TaqMan RNaseP detection assay) on a StepOnePlus instrument (both Thermo Fisher Scientific). Samples were amplified using a custom-designed gene panel for BC^50^, covering the most recurrent mutations^51^. Library preparation and sequencing were performed using multiplex PCR-based Ion Torrent AmpliSeq (Thermo Fisher Scientific) and Ion S5XL technology, as previously described^52^.

### Bulk RNA-seq of CDOs

CTCs were collected, washed in PBS and lysed in RNA lysis buffer. RNA was extracted using a PicoPure RNA isolation kit following the manufacturer’s instructions. RNA concentration and quality were assessed by Bioanalyzer (Agilent). Libraries were prepared using 5 ng of total RNA with an NEBNext Single Cell/Low Input RNA Library Prep kit (New England Biolabs) following the manufacturer’s instructions. Library concentration was quantified with QuBit, and library size distribution was assessed by Bioanalyzer. Up to 15 libraries were pooled equimolarly and sequenced on a NovaSeq 6000 S1 (paired-end, 150 base pairs).

### Bulk RNA-seq analysis

Bcl2fastq2 2.20 was used for conversion. Reads were trimmed for adapter sequences and aligned to the 1000 Genomes Phase 2 assembly of the Genome Reference Consortium human genome (build 37, version hs37d5) with STAR^53^ (v2.5.3a) using the following parameters: alignIntronMax: 500,000; alignMatesGapMax: 500,000; outSAMunmapped: within; outFilterMultimapNmax: 1; outFilterMismatchNmax: 3; outFilterMismatchNoverLmax: 0.3; sjdbOverhang: 50; chimSegmentMin: 15; chimScoreMin: 1; chimScoreJunctionNonGTAG: 0 and chimJunctionOverhangMin: 15. GENCODE gene annotation (GENCODE release 19) was used for building the index. BAM files were sorted using SAMtools^54^ (v1.6), and duplicates were marked with Sambamba^55^ (v0.6.5). Raw counts were generated using featureCounts^56^ (Subread version 1.5.3).

For calculation of normalized counts, mitochondrial RNA, tRNA, rRNA and all transcripts from the Y and X chromosomes were removed, and normalization was performed in analogy to transcripts per million.

Differential gene expression analysis was performed using DESeq2 (ref. ^56^; v1.26.0). The lfcshrink function was used to define differentially expressed genes (| log_2_ (fold change) |) of ≥1, adjusted *P* value of ≤0.05). The log_2_ (fold change) values (nonshrinked) were used for GSEA with clusterProfiler^57^ and the Molecular Signatures Database v7.411 as reference gene sets. Data handling was performed in R (v3.6.0) using RStudio (v1.4).

### Gene expression analysis of human CTCs: scRNA-seq

From cryopreserved vials of CTC samples, single live Lin^–^EpCAM^+^ cells were directly sorted by FACS into 100 µl of TRIzol (Thermo Fisher). Samples were immediately snap-frozen in liquid nitrogen and stored at −80 °C. For RNA isolation, TRIzol samples were thawed on ice and mixed with 20 µl of chloroform. After incubation at room temperature for 3 min, samples were centrifuged (12,000*g*, 5 min, room temperature) and immediately transferred on ice. The aqueous phase was collected and mixed with 0.4 µl of GlycoBlue (Thermo Fisher) in 75 µl of isopropanol, and samples were stored at −20 °C for at least 5 days. Samples were centrifuged (13,000*g*, 1 h, 4 °C), and the pellet was washed with 70% ethanol and centrifuged again (13,000*g*, 15 min, 4 °C). The pellet was resuspended in 5 µl of Smart-Seq2 buffer. Whole-transcriptome amplification was performed using the modified Smart-Seq2 protocol as previously described^58^. Libraries were constructed using a Nextera XT DNA Library Preparation kit (Illumina) according to the manufacturer’s instructions but using one-fourth of all volumes. Sequencing was performed on an Illumina HiSeq 2500 platform.

### scRNA-seq analysis

Raw data processing was performed with kallisto^59^ (v0.43.0). The kallisto index file was generated with a hg38 transcriptome fasta file (release-98) downloaded from Ensembl, and reads were then pseudoaligned to the transcriptome with kallisto in quant mode. The R package tximport^60^ (v1.14.2) was used to perform gene-level summaries, and the resulting count matrix was imported as a SingleCellExperiment object in R.

The R packages scater^61^ (v1.14.6) and scran^62^ (v1.14.6) were used to calculate quality control metrics and remove cells with less than 1 × 10^5^ total counts, less than 2,500 detected features or a percentage of mitochondrial genes higher than 20%. Normalization and log transformation of the data were performed with the functions computeSumFactors and logNormCounts. Cells not expressing the epithelial marker EpCAM or expressing the leukocyte marker CD45 were removed, leaving 318 putative CTCs.

Cells were then separated into *ERBB3*^hi^ and *ERBB3*^lo^ populations based on the results of a *k*-means clustering (*k* = 2) on *ERBB3* expression values (Fig. 3d). To define a HER3 signature, genes that were not expressed in at least 20% of cells were removed. Differentially expressed genes between the *ERBB3*^hi^ and *ERBB3*^lo^ populations were then computed using the pairwiseWilcox function from scran (FDR < 0.1). Resulting significant genes were intersected with genes whose expression showed a significant Pearson correlation (FDR < 0.1) with the expression of *ERBB3* and protein-coding genes, yielding 592 upregulated genes and 6 downregulated genes (Supplementary Table 3). To see if the HER3 signature could be used to separate the *ERBB3*^hi^ and *ERBB3*^lo^ populations in the three individuals, *z* scores were computed for each signature gene for the two populations ($z=\,\frac{x-\,{\mu }}{{\sigma }}$), and the mean over all genes was calculated. For better UMAP visualization and coloring of expression of different genes (Fig. 6a and Extended Data Figs. 6b,c and 10a), data were integrated with mutual nearest neighbors, as implemented in the fastMNN() function from the batchelor^63^ (v1.2.4) R package.

### Genomic analysis of CDOs and participant-matched lesions

Whole-genome sequencing and whole-exome sequencing data were aligned and analyzed using the Sarek^64^ (v3.1.2) Nextflow^65^ (v22.10.7) workflow from the nf-core framework. Briefly, initial quality control and read trimming were performed with FASTQC (v0.11.9) and fastp^66^ (v0.23.2). Trimmed reads were then aligned to the GRCh38 reference genome using BWA-mem^67^ (v0.7.17-r1188). Aligned reads were further preprocessed using GATK4 (ref. ^46^; v4.3.0.0). Variants were called using Strelka2 (ref. ^46^; v2.9.10), Mutect2 or Manta^68^ (v1.6.0) using matching germline controls when possible. Variants were annotated using Ensembl VEP^69^ (v106.1). For subsequent analyses, only variants fulfilling the following criteria were selected: (1) called by both Strelka2 and Mutect2, (2) FILTER = = PASS, (3) at least 20 reads mapped in the germline control sample or in the tumor sample and (4) at least 2 reads mapped in the alternative allele in the tumor sample. Oncoplot was generated using R 4.3.0 and the maftools^70^ package (v2.16.0). A panel of most common and relevant mutated genes was defined based on previous studies on MBCPs^25,30^.

### Gene expression analysis in longitudinal clinical lesions

RNA isolation from fresh-frozen or formalin-fixed paraffin-embedded tumor samples, quality control, stranded library preparation, sequencing, alignment of resulting reads and their summarization were all performed as previously described^25^. The obtained read counts for 22 longitudinally profiled tumor pairs were subjected to variance-stabilizing transformation using DESeq2 (ref. ^71^; v1.22.2), which was subsequently used as input for the PCA. Based on the PCA, the initial set was further reduced to 14 pairs, selecting samples that belong to the same cluster along the first PC axis.

### Genome-wide CRISPRa screen to identify regulators of NRG1 dependency

The human CRISPRa pooled library Set A (Addgene plasmid 92379; a gift from D. Root and J. Doench, Broad Institute of Harvard and MIT) was amplified as previously described^72^. CRISPR library complexity was assessed via next-generation sequencing using a HiSeq2000. For the validation experiments, individual gRNAs were cloned into pXPR_502 (Addgene 96923; a gift from J. Doench and D. Root) lentivector for CRISPRa experiments via restriction digest with BsmBI (New England Biolabs, R0739). CTC596 cells were transduced with lentiviral particles carrying dCas9-VP64 (lenti dCas9VP64_Blast, Addgene plasmid 61425; a gift from F. Zhang, Broad Institute of MIT and Harvard) at a multiplicity of infection of ∼0.6. After recovery, cells were selected with blasticidin (20 μg ml^–1^). CTC596 cells expressing dCas9-VP64 were transduced with the genome-wide Calabrese CRISPRa library in two independent experiments at a multiplicity of infection of ∼0.15. For each replicate, ∼200 million cells were transduced, achieving a representation of 300/500 cells per gRNA. After initial recovery for 2 days, cells were selected with puromycin (1 μg ml^–1^) for 4 days. For each replicate, cells were collected at *t*_0_ to determine baseline gRNA representation, *t*_1_ and *t*_2_. Genomic DNA was extracted using a Quick-DNA Midiprep Plus kit (Zymo Research, D4075) following the manufacturer’s instructions, and next-generation sequencing libraries were prepared as previously described^72^. Libraries were sequenced as a multiplexed pool on a HiSeq2000 (125 cycles read 1, 8 cycles index 1 (i7)). Data were analyzed using the PinAPL-Py web tool^73^. Briefly, after trimming of the adapters, reads were aligned to the reference library and counted. A gene score (SigmaFC) was then calculated by taking the sum of the log (fold change) values of all sgRNAs targeting that gene and multiplying it by the number of its sgRNAs that reached statistically significant enrichment. The Benjamini–Hochberg correction method was used for *P* value correction.

### Statistics and reproducibility

Sample sizes are similar to those reported in previous publications^19,74,75^, and no statistical method was used to predetermine sample size. Statistical analysis and data visualization were performed using GraphPad Prism (v10.3.0 and earlier) software, except for genomic and transcriptomic analysis and visualization, which were performed using R. Data collection and analysis were not performed blind to the conditions of the experiments. For in vivo treatment studies, mice were randomized before the start of treatment to ensure that each group started with an approximately equal mean tumor size. For comparison between two sample groups, statistical analysis was conducted using a two-tailed (paired or unpaired) Student’s *t*-test, two-tailed Mann–Whitney test and two-tailed Wilcoxon test, unless otherwise stated. For multiple comparisons, one- or two-way ANOVA was used. Data distribution was not formally tested. A *P* value of <0.05 was used as a cutoff for significance. Data are generally presented as mean ± s.e.m. of *n* = *x* experiments, with *x* indicating the number of independent experiments performed, which is noted in the figure legends.

### Reporting summary

Further information on research design is available in the Nature Portfolio Reporting Summary linked to this article.

## Supplementary information

Supplementary Information Supplementary Fig. 1.

Reporting Summary

Supplementary Tables 1–5 Supplementary Table 1. Participant and tumor characteristics. Supplementary Table 2. Genomic analysis of CDOs and matched lesions. Supplementary Table 3. HER3 CTC signature from scRNA-seq analysis of CTC250, CTC595 and CTC596 cells. Supplementary Table 4. Genome-wide CRISPRa screen. Supplementary Table 5. Oligonucleotides.

## Source data

Source Data Figs. 1–6 and Extended Data Figs. 3, 5, 7, 9 and 10 Statistical source data.

Source Data Figs. 1 and 3–5 and Extended Data Fig. 3 Unprocessed western blots.

## Data Availability

DNA-seq, RNA-seq, scRNA-seq and microarray data supporting the findings of this study have been deposited in the European Genome–Phenome Archive (EGA) under study number EGAS00001007582. Access to these data is restricted to protect participant information. Researchers can apply for access through the EGA Data Access Request, which will be reviewed by the Data Access Committee and Data Protection Office. A Data Transfer Agreement must be signed by both parties, ensuring that data confidentiality is maintained and the data are used for the stated research purpose. The access process can take up to several weeks. Further information about the EGA can be found at https://ega-archive.org. The human BC reverse-phase protein assay data were derived from the TCGA Research Network (http://cancergenome.nih.gov/) and analyzed using Kaplan–Meier Plotter (https://kmplot.com/). The gene expression profiles of commercially available BC and acute myeloid leukemia cell lines were derived from the Cancer Cell Line Encyclopedia dataset (PRJNA523380). The drug sensitivity and expression data on commercially available datasets were retrieved from the DepMap portal (CTDv2 database; https://depmap.org/portal/ccle/). Genome-wide CRISPRa screen data are available in Supplementary Table 4. Source data are provided with this paper. All other data supporting the findings of this study are available from the corresponding authors on reasonable request.

## Code availability

All relevant package and software information is provided in the Methods. No custom code was generated during the course of this study.

## References

[1] Baccelli, I. et al. Identification of a population of blood circulating tumor cells from breast cancer patients that initiates metastasis in a xenograft assay. *Nat. Biotechnol.* **31**, 539–544 (2013). 23609047 10.1038/nbt.2576

[2] Janni, W. J. et al. Pooled analysis of the prognostic relevance of circulating tumor cells in primary breast cancer. *Clin. Cancer Res.* **22**, 2583–2593 (2016). 26733614 10.1158/1078-0432.CCR-15-1603

[3] Alix-Panabieres, C. & Pantel, K. Clinical applications of circulating tumor cells and circulating tumor DNA as liquid biopsy. *Cancer Discov.* **6**, 479–491 (2016). 26969689 10.1158/2159-8290.CD-15-1483

[4] Gkountela, S. et al. Circulating tumor cell clustering shapes DNA methylation to enable metastasis seeding. *Cell* **176**, 98–112 (2019). 30633912 10.1016/j.cell.2018.11.046 PMC6363966

[5] Koch, C. et al. Characterization of circulating breast cancer cells with tumorigenic and metastatic capacity. *EMBO Mol. Med.* **12**, e11908 (2020). 32667137 10.15252/emmm.201911908 PMC7507517

[6] Zhang, L. et al. The identification and characterization of breast cancer CTCs competent for brain metastasis. *Sci. Transl. Med.* **5**, 180ra148 (2013). 10.1126/scitranslmed.3005109 PMC3863909 23576814

[7] Yu, M. et al. Ex vivo culture of circulating breast tumor cells for individualized testing of drug susceptibility. *Science* **345**, 216–220 (2014). 25013076 10.1126/science.1253533 PMC4358808

[8] Eslami, S. Z., Cortes-Hernandez, L. E., Thomas, F., Pantel, K. & Alix-Panabieres, C. Functional analysis of circulating tumour cells: the KEY to understand the biology of the metastatic cascade. *Br. J. Cancer* **127**, 800–810 (2022). 35484215 10.1038/s41416-022-01819-1 PMC9427839

[9] Alix-Panabieres, C. & Pantel, K. Liquid biopsy: from discovery to clinical application. *Cancer Discov.* **11**, 858–873 (2021). 33811121 10.1158/2159-8290.CD-20-1311

[10] Yu, M. et al. Circulating breast tumor cells exhibit dynamic changes in epithelial and mesenchymal composition. *Science* **339**, 580–584 (2013). 23372014 10.1126/science.1228522 PMC3760262

[11] Perez-Gonzalez, A., Bevant, K. & Blanpain, C. Cancer cell plasticity during tumor progression, metastasis and response to therapy. *Nat. Cancer* **4**, 1063–1082 (2023). 37537300 10.1038/s43018-023-00595-y PMC7615147

[12] Dujon, A. M. et al. Is there one key step in the metastatic cascade? *Cancers* **13**, 3693 (2021). 34359593 10.3390/cancers13153693 PMC8345184

[13] Jordan, N. V. et al. HER2 expression identifies dynamic functional states within circulating breast cancer cells. *Nature* **537**, 102–106 (2016). 27556950 10.1038/nature19328 PMC5161614

[14] Schettini, F. et al. Clinical, pathological, and *PAM50* gene expression features of HER2-low breast cancer. *NPJ Breast Cancer* **7**, 1 (2021). 33397968 10.1038/s41523-020-00208-2 PMC7782714

[15] Modi, S. et al. Trastuzumab deruxtecan in previously treated HER2-low advanced breast cancer. *N. Engl. J. Med.* **387**, 9–20 (2022). 35665782 10.1056/NEJMoa2203690 PMC10561652

[16] Lee-Hoeflich, S. T. et al. A central role for HER3 in *HER2*-amplified breast cancer: implications for targeted therapy. *Cancer Res.* **68**, 5878–5887 (2008). 18632642 10.1158/0008-5472.CAN-08-0380

[17] Berdiel-Acer, M. et al. Stromal NRG1 in luminal breast cancer defines pro-fibrotic and migratory cancer-associated fibroblasts. *Oncogene* **40**, 2651–2666 (2021). 33692466 10.1038/s41388-021-01719-3 PMC8049869

[18] Zhang, Z. et al. Tumor microenvironment-derived NRG1 promotes antiandrogen resistance in prostate cancer. *Cancer Cell* **38**, 279–296 (2020). 32679108 10.1016/j.ccell.2020.06.005 PMC7472556

[19] Saini, M. et al. Resistance to mesenchymal reprogramming sustains clonal propagation in metastatic breast cancer. *Cell Rep.* **42**, 112533 (2023). 37257449 10.1016/j.celrep.2023.112533

[20] Linnemann, J. R. et al. Quantification of regenerative potential in primary human mammary epithelial cells. *Development* **142**, 3239–3251 (2015). 26071498 10.1242/dev.123554 PMC4582177

[21] Nagashima, T. et al. Quantitative transcriptional control of ERBB receptor signaling undergoes graded to biphasic response for cell differentiation. *J. Biol. Chem.* **282**, 4045–4056 (2007). 17142811 10.1074/jbc.M608653200

[22] Sachs, N. et al. A living biobank of breast cancer organoids captures disease heterogeneity. *Cell* **172**, 373–386 (2018). 29224780 10.1016/j.cell.2017.11.010

[23] Dekkers, J. F. et al. Long-term culture, genetic manipulation and xenotransplantation of human normal and breast cancer organoids. *Nat. Protoc.* **16**, 1936–1965 (2021). 33692550 10.1038/s41596-020-00474-1 PMC8221035

[24] Yoshida, Y., Takahashi, K., Okita, K., Ichisaka, T. & Yamanaka, S. Hypoxia enhances the generation of induced pluripotent stem cells. *Cell Stem Cell* **5**, 237–241 (2009). 19716359 10.1016/j.stem.2009.08.001

[25] Hlevnjak, M. et al. CATCH: a prospective precision oncology trial in metastatic breast cancer. *JCO Precis Oncol.* **5**, PO.20.00248 (2021). 10.1200/PO.20.00248 PMC8140780 34036222

[26] Cancer Genome Atlas Network. Comprehensive molecular portraits of human breast tumours. *Nature* **490**, 61–70 (2012). 23000897 10.1038/nature11412 PMC3465532

[27] Ros, S. et al. Metabolic imaging detects resistance to PI3Kα inhibition mediated by persistent FOXM1 expression in ER^+^ breast cancer. *Cancer Cell* **38**, 516–533 (2020). 32976773 10.1016/j.ccell.2020.08.016 PMC7562820

[28] Narayan, P. et al. FDA approval summary: alpelisib plus fulvestrant for patients with HR-positive, HER2-negative, PIK3CA-mutated, advanced or metastatic breast cancer. *Clin. Cancer Res.* **27**, 1842–1849 (2021). 33168657 10.1158/1078-0432.CCR-20-3652 PMC8535764

[29] Osz, A., Lanczky, A. & Gyorffy, B. Survival analysis in breast cancer using proteomic data from four independent datasets. *Sci. Rep.* **11**, 16787 (2021). 34408238 10.1038/s41598-021-96340-5 PMC8373859

[30] Li, Q. et al. INK4 tumor suppressor proteins mediate resistance to CDK4/6 kinase inhibitors. *Cancer Discov.* **12**, 356–371 (2022). 34544752 10.1158/2159-8290.CD-20-1726 PMC8831444

[31] Gavine, P. R. et al. AZD4547: an orally bioavailable, potent, and selective inhibitor of the fibroblast growth factor receptor tyrosine kinase family. *Cancer Res.* **72**, 2045–2056 (2012). 22369928 10.1158/0008-5472.CAN-11-3034

[32] Sidaway, P. HER2-targeted agents overcome resistance. *Nat. Rev. Clin. Oncol.* **17**, 133 (2020). 31900443 10.1038/s41571-019-0325-y

[33] Siegel, R. L., Miller, K. D., Fuchs, H. E. & Jemal, A. Cancer statistics, 2022. *CA Cancer J. Clin.* **72**, 7–33 (2022). 35020204 10.3322/caac.21708

[34] Jin, H. et al. EGFR activation limits the response of liver cancer to lenvatinib. *Nature* **595**, 730–734 (2021). 34290403 10.1038/s41586-021-03741-7

[35] Prawira, A., Le, T. B. U., Ho, R. Z. W. & Huynh, H. Upregulation of the ERBB family by EZH2 in hepatocellular carcinoma confers resistance to FGFR inhibitor. *J. Cancer Res. Clin. Oncol.* **147**, 2955–2968 (2021). 34156519 10.1007/s00432-021-03703-6 PMC8397639

[36] Azuma, K. et al. FGFR1 activation is an escape mechanism in human lung cancer cells resistant to afatinib, a pan-EGFR family kinase inhibitor. *Oncotarget* **5**, 5908–5919 (2014). 25115383 10.18632/oncotarget.1866 PMC4171601

[37] Issa, A. et al. Combinatorial targeting of FGF and ERBB receptors blocks growth and metastatic spread of breast cancer models. *Breast Cancer Res.* **15**, R8 (2013). 23343422 10.1186/bcr3379 PMC3672810

[38] Hanker, A. B. et al. HER2-overexpressing breast cancers amplify FGFR signaling upon acquisition of resistance to dual therapeutic blockade of HER2. *Clin. Cancer Res.* **23**, 4323–4334 (2017). 28381415 10.1158/1078-0432.CCR-16-2287 PMC5540793

[39] Coetzee, A. S. et al. Nuclear FGFR1 promotes pancreatic stellate cell-driven invasion through up-regulation of neuregulin 1. *Oncogene* **42**, 491–500 (2023). 36357571 10.1038/s41388-022-02513-5 PMC9918430

[40] Hashimoto, Y. et al. A novel HER3-targeting antibody–drug conjugate, U3-1402, exhibits potent therapeutic efficacy through the delivery of cytotoxic payload by efficient internalization. *Clin. Cancer Res.* **25**, 7151–7161 (2019). 31471314 10.1158/1078-0432.CCR-19-1745

[41] Janne, P. A. et al. Efficacy and safety of patritumab deruxtecan (HER3-DXd) in EGFR inhibitor-resistant, EGFR-mutated non-small cell lung cancer. *Cancer Discov.* **12**, 74–89 (2022). 34548309 10.1158/2159-8290.CD-21-0715 PMC9401524

[42] Oliveira, M. et al. Patritumab deruxtecan in untreated hormone receptor-positive/HER2-negative early breast cancer: final results from part A of the window-of-opportunity SOLTI TOT-HER3 pre-operative study. *Ann. Oncol.* **34**, 670–680 (2023). 37211044 10.1016/j.annonc.2023.05.004

[43] Al-Hajj, M., Wicha, M. S., Benito-Hernandez, A., Morrison, S. J. & Clarke, M. F. Prospective identification of tumorigenic breast cancer cells. *Proc. Natl Acad. Sci. USA* **100**, 3983–3988 (2003). 12629218 10.1073/pnas.0530291100 PMC153034

[44] Schmitt, M. & Pawlita, M. High-throughput detection and multiplex identification of cell contaminations. *Nucleic Acids Res.* **37**, e119 (2009). 19589807 10.1093/nar/gkp581 PMC2764421

[45] Ghandi, M. et al. Next-generation characterization of the Cancer Cell Line Encyclopedia. *Nature* **569**, 503–508 (2019). 31068700 10.1038/s41586-019-1186-3 PMC6697103

[46] McKenna, A. et al. The Genome Analysis Toolkit: a MapReduce framework for analyzing next-generation DNA sequencing data. *Genome Res.* **20**, 1297–1303 (2010). 20644199 10.1101/gr.107524.110 PMC2928508

[47] Heckl, D. et al. Generation of mouse models of myeloid malignancy with combinatorial genetic lesions using CRISPR–Cas9 genome editing. *Nat. Biotechnol.* **32**, 941–946 (2014). 24952903 10.1038/nbt.2951 PMC4160386

[48] Ran, F. A. et al. Genome engineering using the CRISPR–Cas9 system. *Nat. Protoc.* **8**, 2281–2308 (2013). 24157548 10.1038/nprot.2013.143 PMC3969860

[49] Noll, E. M. et al. CYP3A5 mediates basal and acquired therapy resistance in different subtypes of pancreatic ductal adenocarcinoma. *Nat. Med.* **22**, 278–287 (2016). 26855150 10.1038/nm.4038 PMC4780258

[50] Pfarr, N. et al. Targeted next-generation sequencing enables reliable detection of HER2 (ERBB2) status in breast cancer and provides ancillary information of clinical relevance. *Genes Chromosomes Cancer* **56**, 255–265 (2017). 27792260 10.1002/gcc.22431

[51] Kriegsmann, M. et al. Mutational profiles in triple-negative breast cancer defined by ultradeep multigene sequencing show high rates of PI3K pathway alterations and clinically relevant entity subgroup specific differences. *Oncotarget* **5**, 9952–9965 (2014). 25296970 10.18632/oncotarget.2481 PMC4259450

[52] Konukiewitz, B. et al. Pancreatic neuroendocrine carcinomas reveal a closer relationship to ductal adenocarcinomas than to neuroendocrine tumors G3. *Hum. Pathol.* **77**, 70–79 (2018). 29596894 10.1016/j.humpath.2018.03.018

[53] Dobin, A. et al. STAR: ultrafast universal RNA-seq aligner. *Bioinformatics* **29**, 15–21 (2013). 23104886 10.1093/bioinformatics/bts635 PMC3530905

[54] Li, H. et al. The Sequence Alignment/Map format and SAMtools. *Bioinformatics* **25**, 2078–2079 (2009). 19505943 10.1093/bioinformatics/btp352 PMC2723002

[55] Tarasov, A., Vilella, A. J., Cuppen, E., Nijman, I. J. & Prins, P. Sambamba: fast processing of NGS alignment formats. *Bioinformatics* **31**, 2032–2034 (2015). 25697820 10.1093/bioinformatics/btv098 PMC4765878

[56] Liao, Y., Smyth, G. K. & Shi, W. featureCounts: an efficient general purpose program for assigning sequence reads to genomic features. *Bioinformatics* **30**, 923–930 (2014). 24227677 10.1093/bioinformatics/btt656

[57] Yu, G., Wang, L. G., Han, Y. & He, Q. Y. clusterProfiler: an R package for comparing biological themes among gene clusters. *OMICS* **16**, 284–287 (2012). 22455463 10.1089/omi.2011.0118 PMC3339379

[58] Velten, L. et al. Human haematopoietic stem cell lineage commitment is a continuous process. *Nat. Cell Biol.* **19**, 271–281 (2017). 28319093 10.1038/ncb3493 PMC5496982

[59] Bray, N. L., Pimentel, H., Melsted, P. & Pachter, L. Near-optimal probabilistic RNA-seq quantification. *Nat. Biotechnol.* **34**, 525–527 (2016). 27043002 10.1038/nbt.3519

[60] Soneson, C., Love, M. I. & Robinson, M. D. Differential analyses for RNA-seq: transcript-level estimates improve gene-level inferences. *F1000Res* **4**, 1521 (2015). 26925227 10.12688/f1000research.7563.1 PMC4712774

[61] McCarthy, D. J., Campbell, K. R., Lun, A. T. & Wills, Q. F. Scater: pre-processing, quality control, normalization and visualization of single-cell RNA-seq data in R. *Bioinformatics* **33**, 1179–1186 (2017). 28088763 10.1093/bioinformatics/btw777 PMC5408845

[62] Lun, A. T., McCarthy, D. J. & Marioni, J. C. A step-by-step workflow for low-level analysis of single-cell RNA-seq data with Bioconductor. *F1000Res* **5**, 2122 (2016). 27909575 10.12688/f1000research.9501.1 PMC5112579

[63] Haghverdi, L., Lun, A. T. L., Morgan, M. D. & Marioni, J. C. Batch effects in single-cell RNA-sequencing data are corrected by matching mutual nearest neighbors. *Nat. Biotechnol.* **36**, 421–427 (2018). 29608177 10.1038/nbt.4091 PMC6152897

[64] Garcia, M. et al. Sarek: a portable workflow for whole-genome sequencing analysis of germline and somatic variants. *F1000Res* **9**, 63 (2020). 32269765 10.12688/f1000research.16665.1 PMC7111497

[65] Di Tommaso, P. et al. Nextflow enables reproducible computational workflows. *Nat. Biotechnol.* **35**, 316–319 (2017). 28398311 10.1038/nbt.3820

[66] Chen, S., Zhou, Y., Chen, Y. & Gu, J. fastp: an ultra-fast all-in-one FASTQ preprocessor. *Bioinformatics* **34**, i884–i890 (2018). 30423086 10.1093/bioinformatics/bty560 PMC6129281

[67] Li, H. & Durbin, R. Fast and accurate short read alignment with Burrows–Wheeler transform. *Bioinformatics* **25**, 1754–1760 (2009). 19451168 10.1093/bioinformatics/btp324 PMC2705234

[68] Chen, X. et al. Manta: rapid detection of structural variants and indels for germline and cancer sequencing applications. *Bioinformatics* **32**, 1220–1222 (2016). 26647377 10.1093/bioinformatics/btv710

[69] McLaren, W. et al. The Ensembl variant effect predictor. *Genome Biol.* **17**, 122 (2016). 27268795 10.1186/s13059-016-0974-4 PMC4893825

[70] Mayakonda, A., Lin, D. C., Assenov, Y., Plass, C. & Koeffler, H. P. Maftools: efficient and comprehensive analysis of somatic variants in cancer. *Genome Res.* **28**, 1747–1756 (2018). 30341162 10.1101/gr.239244.118 PMC6211645

[71] Love, M. I., Huber, W. & Anders, S. Moderated estimation of fold change and dispersion for RNA-seq data with DESeq2. *Genome Biol.* **15**, 550 (2014). 25516281 10.1186/s13059-014-0550-8 PMC4302049

[72] Joung, J. et al. Genome-scale CRISPR–Cas9 knockout and transcriptional activation screening. *Nat. Protoc.* **12**, 828–863 (2017). 28333914 10.1038/nprot.2017.016 PMC5526071

[73] Spahn, P. N. et al. PinAPL-Py: a comprehensive web-application for the analysis of CRISPR/Cas9 screens. *Sci. Rep.* **7**, 15854 (2017). 29158538 10.1038/s41598-017-16193-9 PMC5696473

[74] Espinet, E. et al. Aggressive PDACs show hypomethylation of repetitive elements and the execution of an intrinsic IFN program linked to a ductal cell of origin. *Cancer Discov.* **11**, 638–659 (2021). 33060108 10.1158/2159-8290.CD-20-1202 PMC9216338

[75] Alborzinia, H. et al. MYCN mediates cysteine addiction and sensitizes neuroblastoma to ferroptosis. *Nat. Cancer* **3**, 471–485 (2022). 35484422 10.1038/s43018-022-00355-4 PMC9050595

