# Supplementary material for: Circulating tumor cell plasticity determines breast cancer therapy resistance via neuregulin 1–HER3 signaling
Source: Nat Cancer. 2025 Jan 3;6(1):67–85. doi: 10.1038/s43018-024-00882-2 (PMC11779641; doi:10.1038/s43018-024-00882-2)

nature cancer

Supplementary information https://doi.org/10.1038/s43018-024-00882-2

Circulating tumor cell plasticity determines breast cancer therapy resistance via neuregulin 1–HER3 signaling

In the format provided by the authors and unedited

Suppl.Fig.1
Gating strategy to detect and isolate live Blood lineage⁻/EpCAM⁺ cells by FACS.

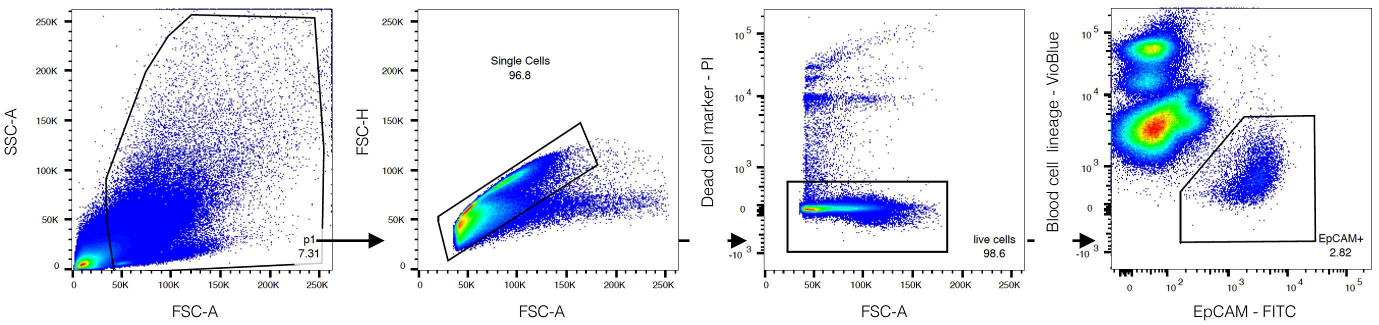

Supplement: Supplementary file 1 — Supplementary Fig. 1. [file 43018_2024_882_MOESM1_ESM.pdf]
