# Supplementary material for: Circulating tumor cell plasticity determines breast cancer therapy resistance via neuregulin 1–HER3 signaling
Source: Nat Cancer. 2025 Jan 3;6(1):67–85. doi: 10.1038/s43018-024-00882-2 (PMC11779641; doi:10.1038/s43018-024-00882-2)
Supplement: Supplementary file 2 — Reporting Summary [file 43018_2024_882_MOESM2_ESM.pdf]

nature portfolio

Corresponding author(s): Andreas Trumpp
Roberto Würth

Last updated by author(s): 2024/09/25

Reporting Summary

Nature Portfolio wishes to improve the reproducibility of the work that we publish. This form provides structure for consistency and transparency in reporting. For further information on Nature Portfolio policies, see our Editorial Policies and the Editorial Policy Checklist.

Statistics

For all statistical analyses, confirm that the following items are present in the figure legend, table legend, main text, or Methods section.

n/a	Confirmed	
☐	☒	The exact sample size (*n*) for each experimental group/condition, given as a discrete number and unit of measurement
☐	☒	A statement on whether measurements were taken from distinct samples or whether the same sample was measured repeatedly
☐	☒	The statistical test(s) used AND whether they are one- or two-sided<br>*Only common tests should be described solely by name; describe more complex techniques in the Methods section.*
☒	☐	A description of all covariates tested
☐	☒	A description of any assumptions or corrections, such as tests of normality and adjustment for multiple comparisons
☐	☒	A full description of the statistical parameters including central tendency (e.g. means) or other basic estimates (e.g. regression coefficient) AND variation (e.g. standard deviation) or associated estimates of uncertainty (e.g. confidence intervals)
☐	☒	For null hypothesis testing, the test statistic (e.g. *F*, *t*, *r*) with confidence intervals, effect sizes, degrees of freedom and *P* value noted<br>*Give P values as exact values whenever suitable.*
☒	☐	For Bayesian analysis, information on the choice of priors and Markov chain Monte Carlo settings
☒	☐	For hierarchical and complex designs, identification of the appropriate level for tests and full reporting of outcomes
☐	☒	Estimates of effect sizes (e.g. Cohen's *d*, Pearson's *r*), indicating how they were calculated

Our web collection on statistics for biologists contains articles on many of the points above.

Software and code

Policy information about availability of computer code

Data collection

Our study uses data output by illumina sequencers and Affy Human U133Plus 2.0 generated at the German Cancer Research Center (DKFZ, Heidelberg)

Data analysis

Statistical analysis and data visualization were performed using GraphPad Prism software ((v10.3.0 and earlier), except for genomic and transcriptomic analysis and visualization, which were carried out using R.
The computational software used in the study include:
- Bulk RNA-seq analysis
Bcl2fastq2 2.20 was used for conversion. Reads were trimmed for adapter sequences and aligned to the 1000 Genomes Phase 2 assembly of the Genome Reference Consortium human genome (build 37, version hs37d5) with STAR (v2.5.3a) using the following parameters: alignIntronMax: 500.000, alignMatesGapMax: 500.000, outSAMunmapped: Within, outFilterMultimapNmax: 1, outFilterMismatchNmax: 3, outFilterMismatchNoverLmax: 0.3, sjdbOverhang: 50, chimSegmentMin: 15, chimScoreMin: 1, chimScoreJunctionNonGTAG: 0, chimJunctionOverhangMin: 15. GENCODE gene annotation (GENCODE Release 19) was used for building the index. BAM files were sorted using SAMtools (v1.6) and duplicates were marked with Sambamba (v0.6.5). Raw counts were generated using featureCounts (Subread version 1.5.3).
For calculation of normalized counts, mtRNA, tRNA, rRNA as well as all transcripts from the Y- and X-chromosome were removed and subsequently normalization was performed in analogy to TPM (transcripts per million).
Analysis of differential expression was performed using DESeq2 (v1.26.0). The lfcshrink function was used to define differentially expressed genes (abs(log2FC) ≥1, p.adj ≤ 0.05). Log2 fold changes (non-shrinked) were used for GSEA analysis with clusterProfiler and the Molecular Signatures Database v7.411 as reference gene sets. Data handling was performed in R (v3.6.0) using RStudio (v1.4).
- scRNA-seq analysis
Raw data processing was performed with kallisto (v0.43.0). The kallisto index file was generated with a hg38 transcriptome fasta file

(release-98) downloaded from Ensembl, and reads were then pseudoaligned to the transcriptome with kallisto in quant mode. The R package txlmport (v1.14.2) was used to perform gene-level summary, and the resulting count matrix was imported as a SingleCellExperiment object in R. The R packages scater (v1.14.6) and scran (v1.14.6) were used to calculate quality control metrics and remove cells with less than 1e5 total counts, less than 2500 detected features, or a percentage of mitochondrial genes higher than 20%. Normalization and log-transformation of the data was performed with the functions computeSumFactors and logNormCounts. Additionally, cells that did not express the epithelial cell marker EpCAM, or expressed the leukocyte marker CD45 were removed. In the end, 318 putative CTCs from three patients were left. For the three patients 250, 595, and 596, cells were then separated into a HER3 high and a HER3 low population based on the results of a k-means clustering (k=2) on HER3 expression values (Fig. 3d). To define a HER3 signature, genes that were not expressed in at least 20% of cells were removed. Then, differential genes between the HER3 high and the HER3 low population were computed using the pairwiseWilcox function from scran (FDR < 0.1). Resulting significant genes were furthermore intersected with genes whose expression showed a significant Pearson correlation (FDR < 0.1) with the expression of HER3, and protein-coding genes, yielding 592 HER3 upregulated genes, and 6 HER3 downregulated genes (Suppl.Table2). To see if the HER3 signature could be used to separate the HER3 high and low populations in the three patients, z-scores were computed for each signature gene for the two populations, and the mean over all genes was calculated. A t-test was then performed between the z-scores for HER3 high and HER3 low to compute a p-value (Extended Data Fig.6d).
For better UMAP visualization and coloring of expression of different genes (Fig.6a, Extended Data Fig.6b,c and 10b), data of the three patients was integrated with mutual nearest neighbors (MNN) as implemented in the fastMNN() function from the batchelor63 (v1.2.4) R package.
- Whole Genome Sequencing and Whole Exome Sequencing
data were aligned and analyzed using the Sarek (version 3.1.2) Nextflow (version 22.10.7) workflow from the nf-core framework. Briefly, initial quality control and reads trimming was performed with FASTQC (version 0.11.9) and fastp (version 0.23.2). Trimmed reads are then aligned to GRCh38 reference genome using BWA-mem (version 0.7.17-r1188). Aligned reads were further preprocessed using GATK4 (version 4.3.0.0) Best practice and variants were called using Strelka2 (version 2.9.10), Mutect2 or Manta (version 1.6.0) using matching germline control when possible. Variants were annotated using Ensembl VEP (version 106.1). For subsequent analyses, only variants fulfilling the following criteria were selected: 1. called by both Strelka2 and Mutect2, 2. FILTER==PASS, 3. at least 20 reads mapped in the germline control sample or in the tumor sample, 4. at least 2 reads mapping in the alternative allele in the tumor sample. Oncoplot were generated using R 4.3.0 and maftools package (version 2.16.0). A panel of most common and relevant mutated genes was defined based on previous studies on metastatic breast cancer patients.

For manuscripts utilizing custom algorithms or software that are central to the research but not yet described in published literature, software must be made available to editors and reviewers. We strongly encourage code deposition in a community repository (e.g. GitHub). See the Nature Portfolio guidelines for submitting code & software for further information.

Data

Policy information about availability of data

All manuscripts must include a data availability statement. This statement should provide the following information, where applicable:

- Accession codes, unique identifiers, or web links for publicly available datasets
- A description of any restrictions on data availability
- For clinical datasets or third party data, please ensure that the statement adheres to our policy

DNAseq, RNAseq, scRNAseq, and microarray data supporting the findings of this study have been deposited in the European Genome-Phenome Archive (EGA), under the study EGAS00001007582. Access to this data is restricted to protect patient information. Researchers can apply for access through the EGA Data Access Request, which will be reviewed by the Data Access Committee and Data Protection Office. A Data Transfer Agreement (DTA) must be signed by both parties, ensuring data confidentiality is maintained and the data is used for the stated research purpose. The access process can take up to several weeks. Further information about EGA can be found at https://ega-archive.org. The human breast cancer reverse phase-protein assay data were derived from the TCGA Research Network: http://cancergenome.nih.gov/ and analyzed using https://kmplot.com/. The gene expression profile of commercially available breast cancer and acute myeloid leukemia cell lines were derived from the Cancer Cell Line Encyclopedia (CCLE) dataset (PRJNA523380). The drug sensitivity and expression data on commercially available datasets were retrieved from DepMap portal (CTDv2 database) (https://depmap.org/portal/ccle/). Genome-wide CRISPRa screen data are available in supplementary Table3. Source data have been provided as Source Data files. All other data supporting the findings of this study are available from the corresponding author on reasonable request.

Research involving human participants, their data, or biological material

Policy information about studies with human participants or human data. See also policy information about sex, gender (identity/presentation), and sexual orientation and race, ethnicity and racism.

Reporting on sex and gender	Female breast cancer patients.
Reporting on race, ethnicity, or other socially relevant groupings	Patient and tumor characteristics are summarized in Table 1.
Population characteristics	Patient and tumor characteristics are summarized in Table 1.
Recruitment	Liquid biopsy samples (pleural and ascitic effusions and peripheral blood withdrawals) were obtained from metastatic breast cancer patients participating at the CATCH (Comprehensive Assessment of clinical feaTures and biomarkers to identify patients with advanced or metastatic breast Cancer for marker driven trials in Humans) trial at the Division of Gynecologic Oncology, National Center for Tumor Diseases (NCT) Heidelberg (case number S-164/2017). Written informed consent was obtained from all patients.<br>CTC-specific assessments were further approved by the ethical committee of the University of Heidelberg (case numbers S295/2009), and University of Mannheim (2010-024238-46). CTC count within the peripheral blood was measured during the course of the disease. Pleural and ascitic effusions were obtained in routine clinical practice.<br>Metastatic breast cancer patients with high CTC counts within the peripheral blood sample (≥10CTCs/7,5ml) were asked to

	participate in the CTC leukapheresis study. The study was approved by the ethical committee of the University of Heidelberg (case number S-408/2013). All the liquid biopsy samples from metastatic breast cancer patients available between 2017 and 2022 were real-time processed, whenever was possible (available people in the lab), without any apparent bias.
Ethics oversight	Liquid biopsy samples (pleural and ascitic effusions and peripheral blood withdrawals) were obtained from metastatic breast cancer patients participating at the CATCH (Comprehensive Assessment of clinical feaTures and biomarkers to identify patients with advanced or metastatic breast Cancer for marker driven trials in Humans) trial at the Division of Gynecologic Oncology, National Center for Tumor Diseases (NCT) Heidelberg (case number S-164/2017).<br>CTC-specific assessments were further approved by the ethical committee of the University of Heidelberg (case numbers S295/2009), and University of Mannheim (2010-024238-46).<br>Metastatic breast cancer patients with high CTC counts within the peripheral blood sample (≥10CTCs/7,5ml) were asked to participate in the CTC leukapheresis study. The study was approved by the ethical committee of the University of Heidelberg (case number S-408/2013).

Note that full information on the approval of the study protocol must also be provided in the manuscript.

Field-specific reporting

Please select the one below that is the best fit for your research. If you are not sure, read the appropriate sections before making your selection.

☒ Life sciences ☐ Behavioural & social sciences ☐ Ecological, evolutionary & environmental sciences

For a reference copy of the document with all sections, see nature.com/documents/nr-reporting-summary-flat.pdf

Life sciences study design

All studies must disclose on these points even when the disclosure is negative.

Sample size	No statistical method was used to predetermine sample size, but sample sizes are similar to those reported in previous publications. To ensure reproducibility of our experimental findings, we generated replicates wherever possible confirming the reproducibility of the results as detailed in the figure legends.
Data exclusions	In scRNAseq analysis, the R packages scater and scran were used to calculate quality control metrics and remove cells with less than 1e5 total counts, less than 2500 detected features, or a percentage of mitochondrial genes higher than 20%. Normalization and log-transformation of the data was performed with the functions computeSumFactors and logNormCounts. Additionally, cells were removed that did not express the epithelial cell marker EpCAM, or expressed the leukocyte marker CD45, since these cells were attributed to be blood cells rather than real CTCs. In the end, 318 putative CTCs from three patients were left.
Replication	To ensure reproducibility of our experimental findings, we generated replicates wherever possible confirming the reproducibility of the results as detailed in the figure legends.
Randomization	For in vitro experiments, samples were analyzed equally with no subsampling, therefore there was no requirement for randomization. For in vivo treatment studies, mice were randomized before the start of treatment to ensure that each group started with an approximately equal mean tumor size.
Blinding	Data collection and analysis were not performed blind to the conditions of the experiments.

Reporting for specific materials, systems and methods

We require information from authors about some types of materials, experimental systems and methods used in many studies. Here, indicate whether each material, system or method listed is relevant to your study. If you are not sure if a list item applies to your research, read the appropriate section before selecting a response.

Materials & experimental systems

n/a	Involved in the study	
☐	☒	Antibodies
☒	☐	Eukaryotic cell lines
☒	☐	Palaeontology and archaeology
☐	☒	Animals and other organisms
☒	☐	Clinical data
☒	☐	Dual use research of concern
☒	☐	Plants

Methods

n/a	Involved in the study	
☒	☐	ChIP-seq
☐	☒	Flow cytometry
☒	☐	MRI-based neuroimaging

Antibodies

Antibodies used	Western-Blot (dilution 1:1000):<br>HER3/ErbB3 (D22C5) XP® Rabbit mAb #12708 Cell Signaling Technology

	Phospho-HER3/ErbB3 (Tyr1289) (21D3) Rabbit mAb #4791 Cell Signaling Technology<br>FGF Receptor 1 (D8E4) XP® Rabbit mAb #9740 Cell Signaling Technology<br>Phospho-Akt (Ser473) (D9E) XP® Rabbit mAb #4060 Cell Signaling Technology<br>Akt (pan) (C67E7) Rabbit mAb #4691 Cell Signaling Technology<br>Phospho-p44/42 MAPK (Erk1/2) (Thr202/Tyr204) (D13.14.4E) XP® Rabbit mAb #4370 Cell Signaling Technology<br>p44/42 MAPK (Erk1/2) (137F5) Rabbit mAb #4695 Cell Signaling Technology<br>Phospho-FAK (Tyr397) (D20B1) Rabbit mAb #8556 Cell Signaling Technology<br>FAK polyclonal Antibody Rabbit #3285 Cell Signaling Technology<br>GAPDH (14C10) Rabbit mAb #2118 Cell Signaling Technology<br>Monoclonal Anti-α-Tubulin antibody produced in mouse T5168 Sigma-Aldrich<br>FACS/Flow-cytometry:<br>EpCAM -FITC and APC-Vio770 (clone HEA-125, REA764 Miltenyi Biotec 1:50)<br>CD45 VioBlue (REA747 clone, Miltenyi Biotec, 1:50)<br>HER3-PE (66223 clone, FAB3481P, R&D, 1:20)<br>CD31-VioBlue (clone AC128, Miltenyi Biotec, 1:50)<br>CD16-VioBlue (clone REA423, Miltenyi Biotec, 1:50)<br>CD41-VioBlue (clone REA386, Miltenyi Biotec, 1:50)<br>CD235a-VioBlue (clone REA175, Miltenyi Biotec, 1:50)<br>anti-mouse CD45 PacificBlue Biolegend Clone 30-F11, Cat# 103116, 1:1000<br>anti-mouse CD11b PacificBlue Biolegend Clone M1/70, Cat# 101226, 1:2000<br>anti-mouse TER-119/Ly-76 PacificBlue Biolegend Clone TER-119, Cat# 116223, 1:200<br>anti-mouse Ly-6G PacificBlue Biolegend Clone 1A8, Cat# A25985, 1:2000<br>anti-mouse CD31 PacificBlue Biolegend Clone 390, BioLegend Cat#102422, 1:1000<br>anti-mouse H2-k d PacificBlue Biolegend Clone SF1-1.1, Cat# 116629, 1:50<br>DAPI (D1306, Thermo Fisher Scientific, 1:1000)<br>Propidium Iodide (P3566, Thermo Fisher Scientific, 1:1000)<br>Immunohistochemistry:<br>anti-EPCAM Agilent DAKO Clone Ber-EP4, Cat# F086001, 1:100<br>anti-CDH1 Agilent DAKO Clone M3612, 1:30<br>anti-estrogen receptor alpha (ER) Thermo Fisher Clone SP1, Cat# MA5-16362, 1:50<br>anti-human Ki-67 Agilent DAKO Clone Ki-67, Cat# F078801, 1:1000<br>anti-human KRT19 Agilent DAKO Clone RCK108, Cat# M0888, 1:50<br>anti-vimentin Agilent DAKO Clone M0725, Cat# M0725, 1:1000<br>HER3/ErbB3 (D22C5) XP® Rabbit mAb #12708 (1:50) Cell Signaling Technology<br>Immunomagnetic sort:<br>anti-CD45 (#130-045-801), Miltenyi Biotec, 20 uL/10e7 cells<br>anti-CD3 (#130-050-101), Miltenyi Biotec, 20 uL/10e7 cells<br>anti-CD31 (#130-091-935), Miltenyi Biotec, 20 uL/10e7 cells<br>anti-CD16 (#130-045-701), Miltenyi Biotec, 20 uL/10e7 cells<br>anti- CD235a (#130-050-501), Miltenyi Biotec, 20 uL/10e7 cells
Validation	All antibodies were validated for the specific application by the manufacturer and validation data is available on the manufacturer's website. A protein size marker was run on every western blot and the size of the assessed bands was compared to the manufactures information. For IHC analysis, positive and negative tissues were included in the staining run whenever possible. HER3 and FGFR1 antibodies were further validated with KO or overexpressing cells.

Animals and other research organisms

Policy information about studies involving animals; ARRIVE guidelines recommended for reporting animal research, and Sex and Gender in Research

Laboratory animals	Female NOD.Cg-Prkdcscid Il2rgtm1Wjl/SzJ (NSG) mice (at least 6 weeks old) were used in the study.
Wild animals	The study did not involve wild animals.
Reporting on sex	Female mice were used in this study as it breast cancer focused.
Field-collected samples	The study did not include samples collected from the field.
Ethics oversight	Animal care and procedures followed the German legal regulations and were previously approved by the governmental review board of the state of Baden-Württemberg, operated by the local Animal Welfare Office (Regierungspräsidium Karlsruhe) under the license number G-240/11, G-115/17, G-104/22. Mice were housed in individually ventilated cages under temperature and humidity control. Cages contained an enriched environment with bedding material.

Note that full information on the approval of the study protocol must also be provided in the manuscript.

Flow Cytometry

Plots

Confirm that:

☒ The axis labels state the marker and fluorochrome used (e.g. CD4-FITC).

☒ The axis scales are clearly visible. Include numbers along axes only for bottom left plot of group (a 'group' is an analysis of identical markers).

☒ All plots are contour plots with outliers or pseudocolor plots.

☒ A numerical value for number of cells or percentage (with statistics) is provided.

Methodology

Sample preparation	Liquid biopsies or expanded CTCs were stained in a PBS solution containing 1% BSA and 2 mM Ethylenediaminetetraacetic acid (EDTA) using panels with the antibodies listed before. See Method section for details for the preparation of all samples.
Instrument	BD FACSAria™ Fusion I or II Cell Sorter, BD LSRFortessa™ Cell Analyzer
Software	FlowJo, BD FACSDiva
Cell population abundance	Due to limited sample material, post-sorting purities were not re-assessed using flow cytometry. Instead, this was done by gating and quantificatoin of populations using FlowJo.
Gating strategy	FSC-A vs SSC-A was the starting gate wherein debris was excluded. Next, single cells were gated based on the exclusion of outliers in FSC-A vs FSC-H. Viable cells were then gated within this population based on low /negative DAPI (or PI) staining (FSC-A vs DAPI/PI). Finally, the ultimate sorting population were gated based on EpCAM+/CD45-(or blood lineage-).

☒ Tick this box to confirm that a figure exemplifying the gating strategy is provided in the Supplementary Information.
