# Supplementary material for: Circulating tumor cell plasticity determines breast cancer therapy resistance via neuregulin 1–HER3 signaling
Source: Nat Cancer. 2025 Jan 3;6(1):67–85. doi: 10.1038/s43018-024-00882-2 (PMC11779641; doi:10.1038/s43018-024-00882-2)

Unprocessed images of blots

Fig.1j

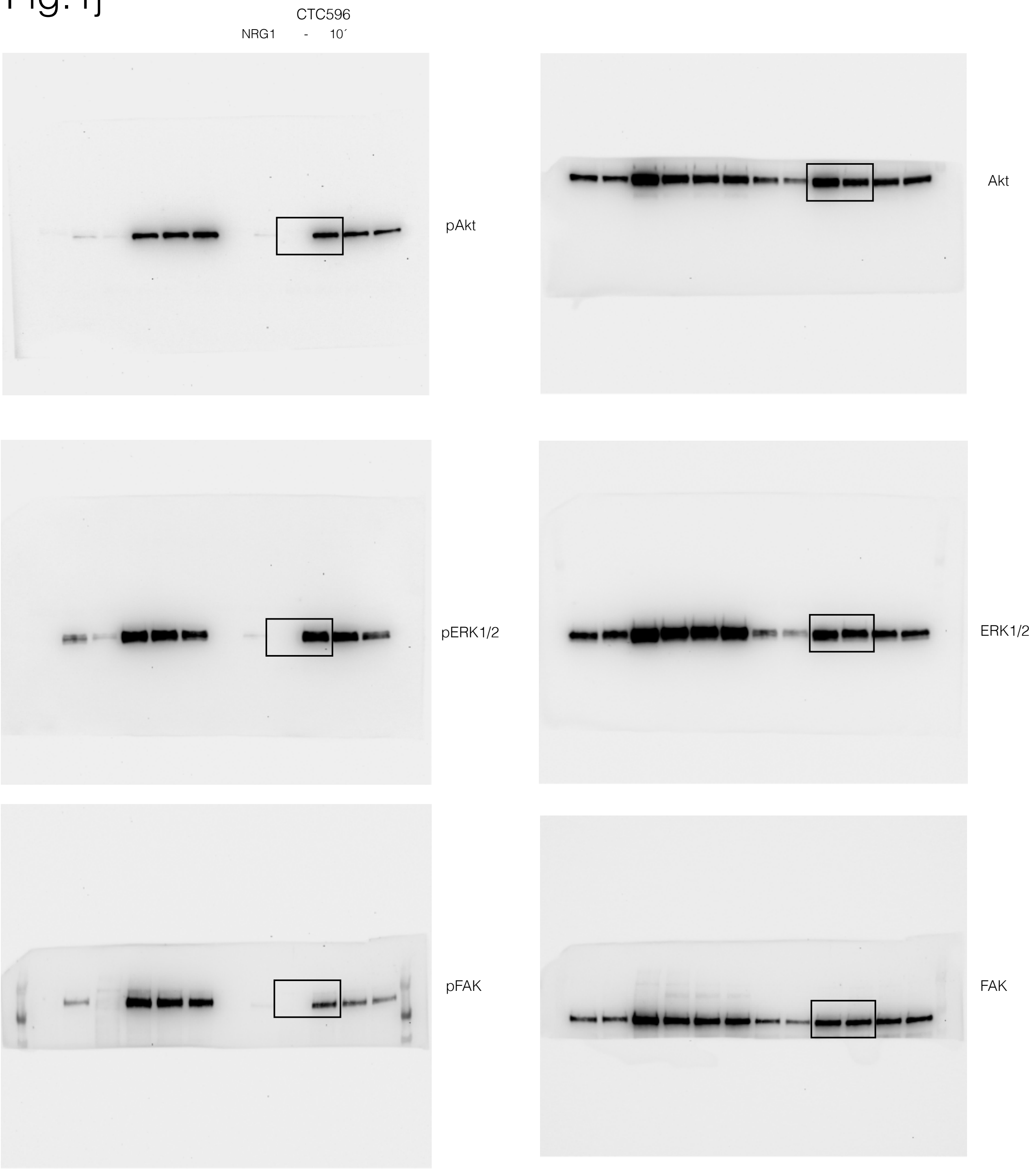

Fig.3f

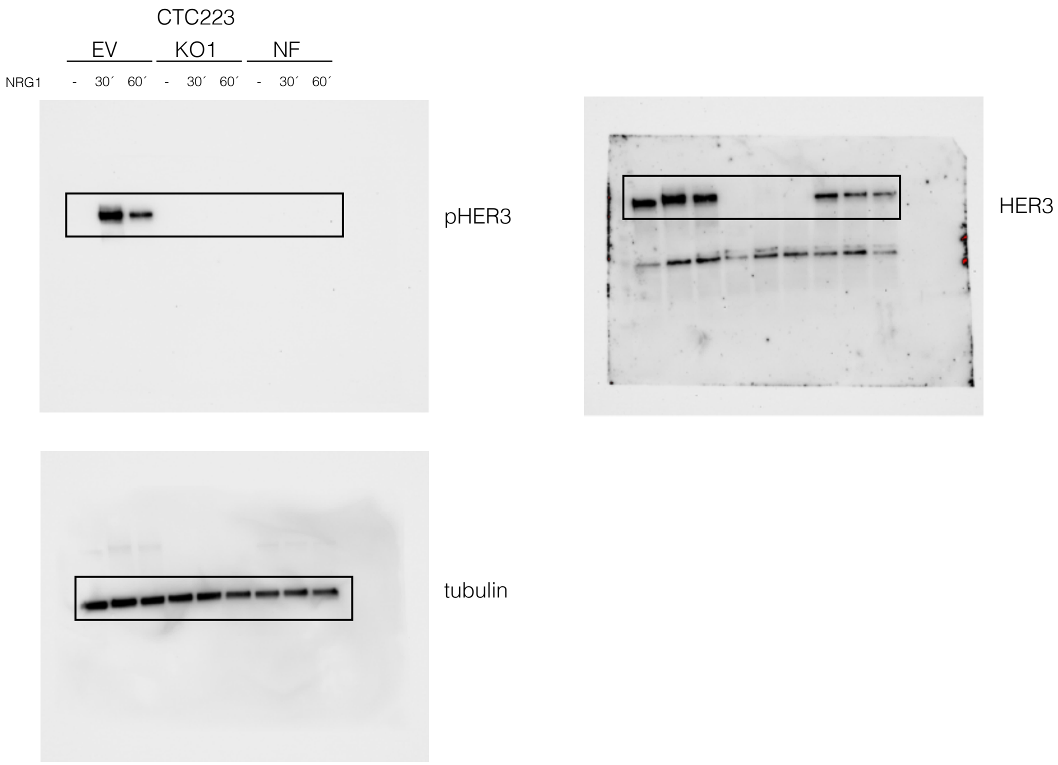

Fig.4e

CTR #1 #2

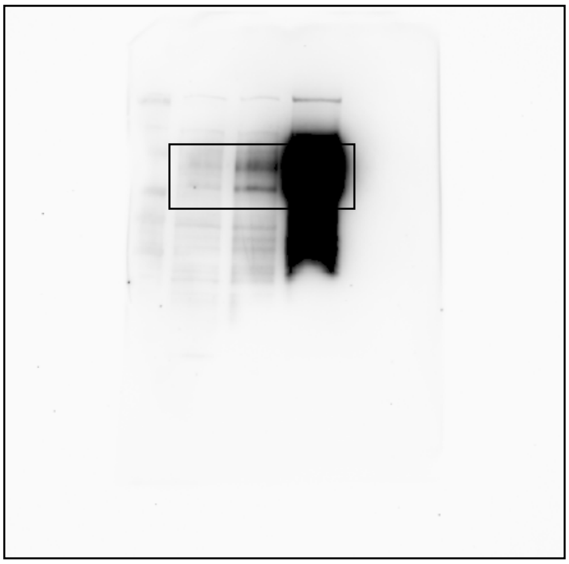

FGFR1

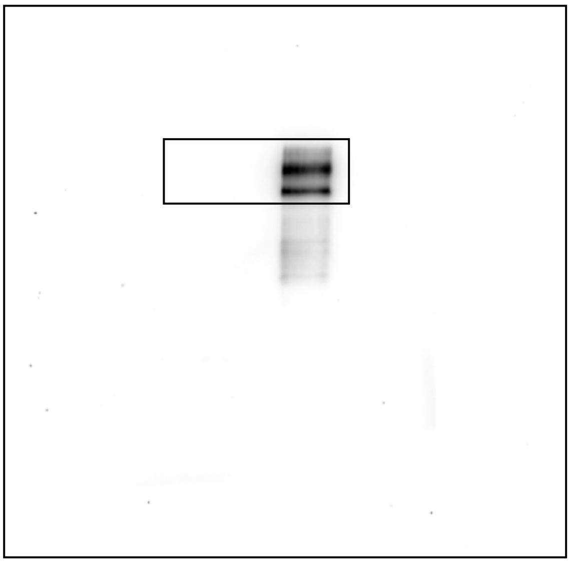

less exp

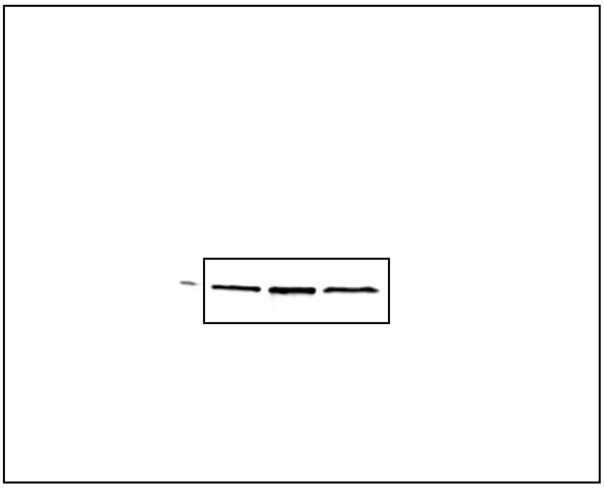

GAPDH

Fig.5d

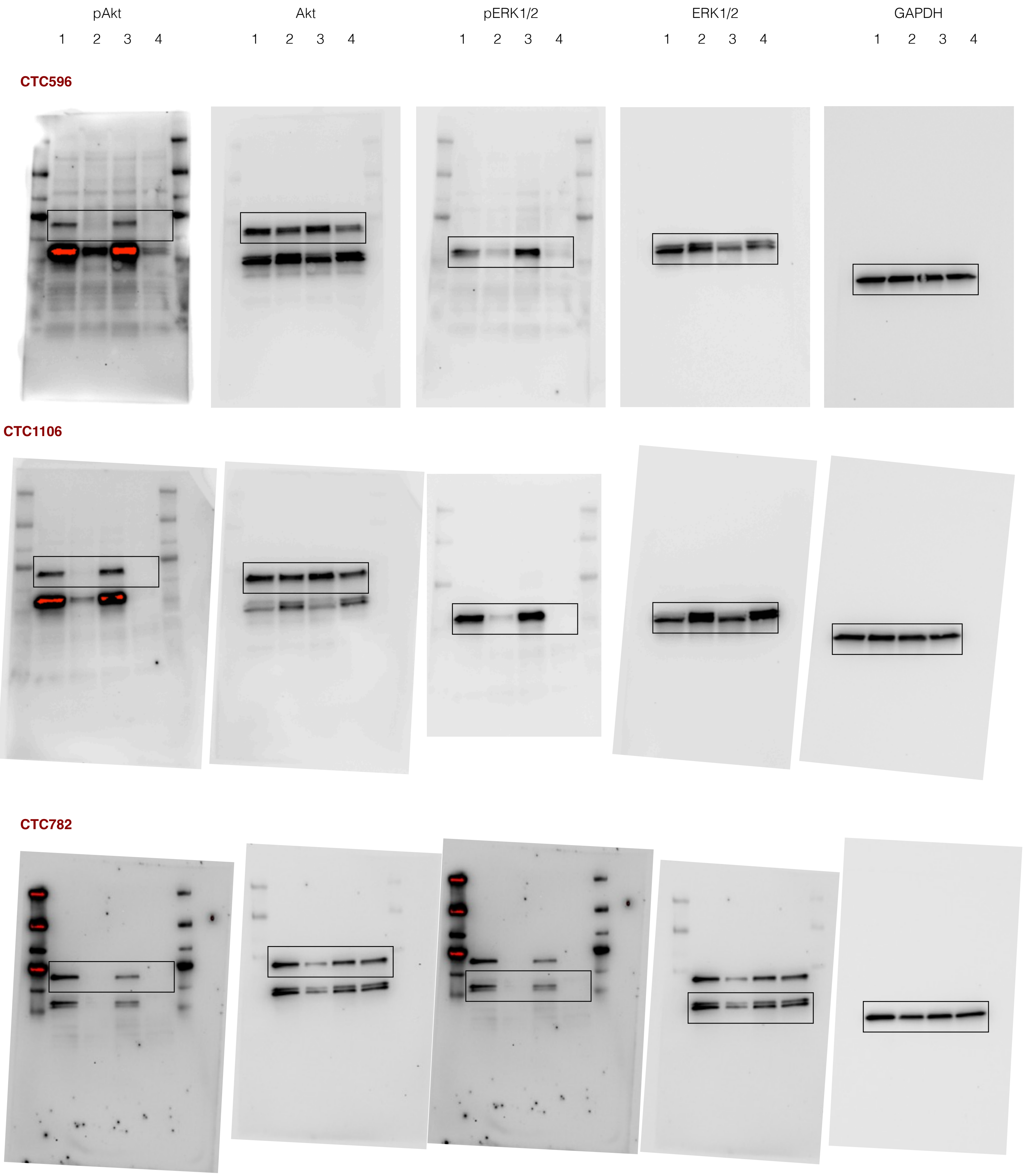

1. DMSO 2. Lapatinib 5uM 3. AZD4547 1uM 4. Lap+AZD NRG1-dependent CDOs

Fig.5d

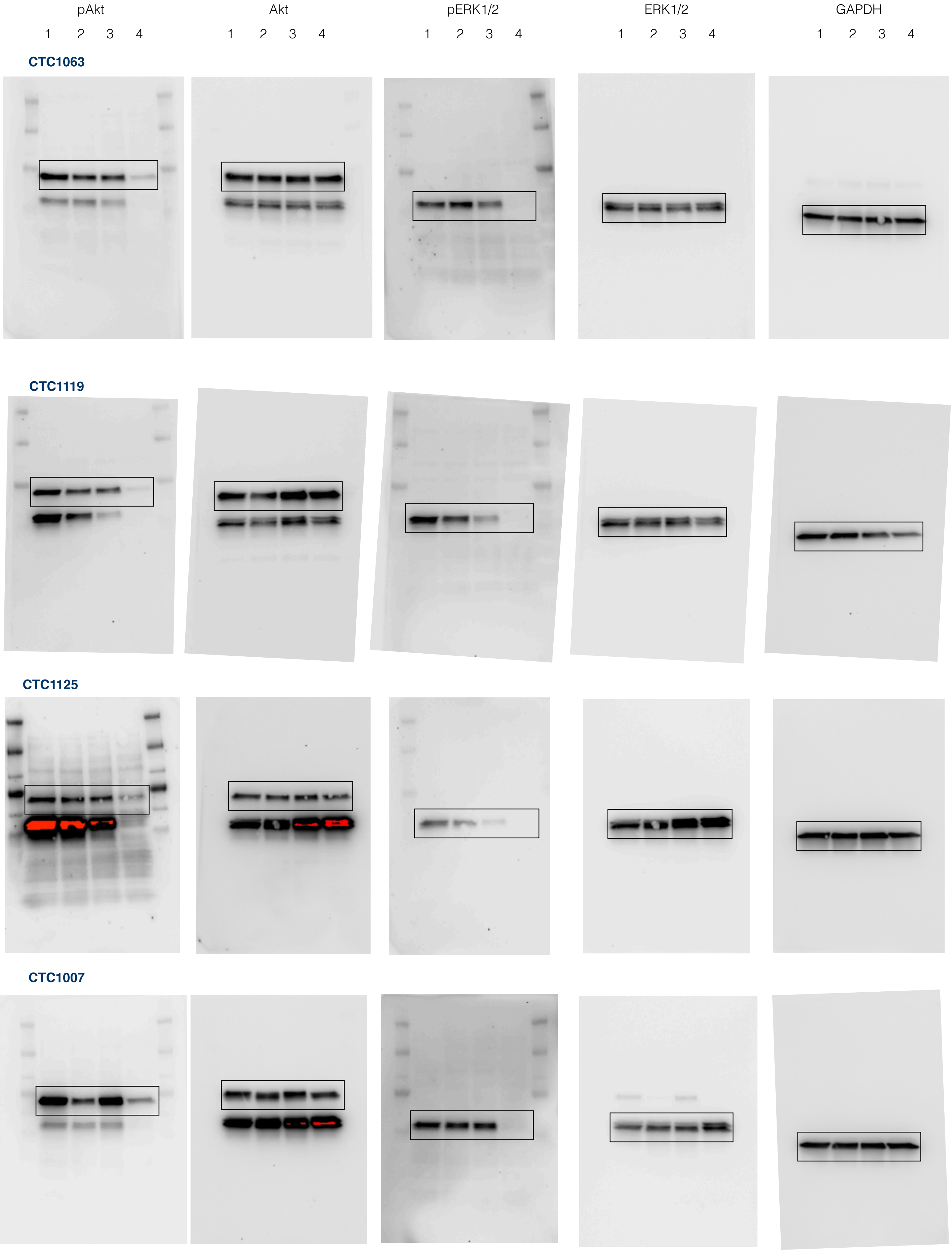

1. DMSO 2. Lapatinib 5uM 3. AZD4547 1uM 4. Lap+AZD

NRG1-independent CDOs

Extended Data Fig.3d

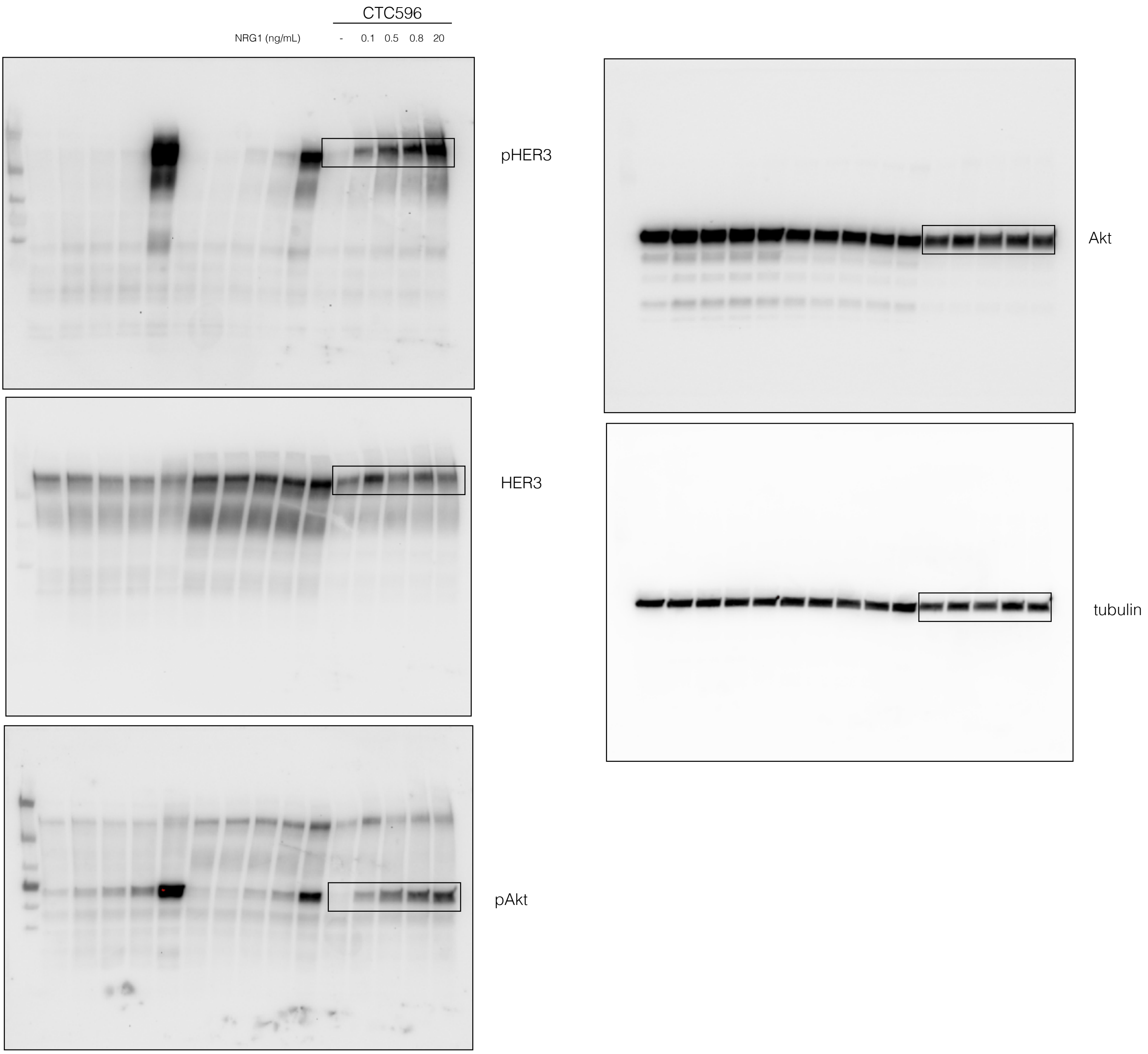

Supplement: Supplementary file 5 — Unprocessed western blots. [file 43018_2024_882_MOESM5_ESM.pdf]
